# A Novel Class of Orally Bioavailable Phenylglycine–Benzoxaborole Conjugates with Antimalarial Activity and Potentially Novel Mechanism of Action

**DOI:** 10.1021/acsmedchemlett.5c00549

**Published:** 2025-12-11

**Authors:** Mokhitli Morake, Dale Taylor, Dina Coertzen, Mathew Njoroge, Liezl Krugmann, Meta Leshabane, Shanté da Rocha, Tarrick Qahash, Gareth Girling, Rachael Coyle, Marcus C. S. Lee, Sergio Wittlin, Manuel Llinás, Lyn-Marie Birkholtz, Gregory S. Basarab, Kelly Chibale

**Affiliations:** † Department of Chemistry, 37716 University of Cape Town, Rondebosch 7701, South Africa; ‡ Drug Discovery and Development Centre (H3D), Institute of Infectious Disease and Molecular Medicine (IDM), 37716 University of Cape Town, Observatory, 7925, South Africa; § Department of Biochemistry, Genetics & Microbiology, Institute for Sustainable Malaria Control, 56410 University of Pretoria, Private Bag X20, Hatfield 0028 Pretoria, South Africa; ∥ Department of Biochemistry and Molecular Biology, The Pennsylvania State University, State College, Pennsylvania 16802, United States of America; ¶ Huck Center for Malaria Research, The Pennsylvania State University, State College, Pennsylvania 16802, United States of America; # 47665 Wellcome Sanger Institute, Wellcome Genome Campus, Hinxton CB10 1SA, United Kingdom; ⊗ Biological Chemistry and Drug Discovery, School of Life Sciences, 3042 University of Dundee, Dundee DD1 4HN, United Kingdom; ○ 30247 Swiss Tropical and Public Health Institute, Socinstrasse 57, 4002 Basel, Switzerland; ● University of Basel, 4003 Basel, Switzerland; ◇ Department of Chemistry, The Pennsylvania State University, State College, Pennsylvania 16802, United States of America; ◆ Drug Discovery and Development Centre (H3D), Department of Chemistry, 37716 University of Cape Town, Rondebosch 7701, South Africa; △ South African Medical Research Council Drug Discovery and Development Research Unit, 37716 University of Cape Town, Rondebosch 7701, South Africa; ▲ Institute of Infectious Disease and Molecular Medicine, 37716 University of Cape Town, Rondebosch 7701, South Africa; †† Department of Biochemistry, Stellenbosch University, Stellenbosch 7600, South Africa

**Keywords:** Benzoxaboroles, CPSF3, structure−activity relationship, microsomal stability

## Abstract

A new class of benzoxaboroles with a phenylglycine appendage was found to display *in vitro* blood stage activity against the human malaria parasite *Plasmodium falciparum* (*Pf*). Structure–activity relationship studies of the starting hit compound **3** resulted in compounds active against *Pf*NF54 drug-sensitive and *Pf*K1 drug-resistant strains with an *in vitro* antiplasmodium IC_50_ < 0.4 μM, selectivity over mammalian cell-lines (selectivity index > 47) and high aqueous solubility (160 to >200 μM). Selected compounds showed good *in vitro* metabolic stability when incubated with human, rat, and mouse liver microsomes and showed no cross-resistance against barcoded mutant lines. Two frontrunner compounds, **6** and **7**, were dosed orally at 50 mg·kg^–1^ using a standard quadrupole dosing regimen in a *P. berghei* mouse infection model and showed encouraging *in vivo* efficacy. This work identifies a promising new class of phenylglycine-based benzoxaboroles, which warrants further medicinal chemistry optimization.

## Graphical abstract

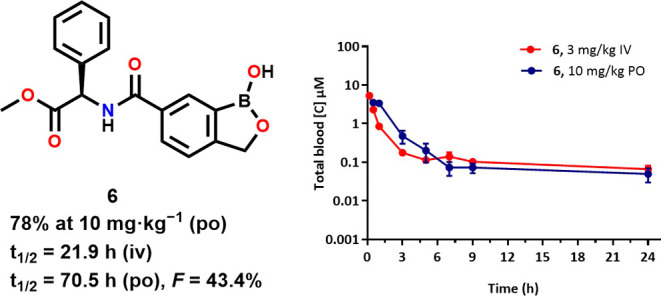

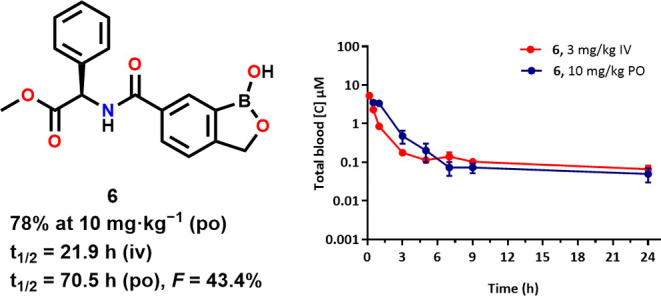

Malaria continues to be a major global health problem with a disproportionate distribution in the impoverished regions of the world. Most cases, particularly in Africa, are due to *Plasmodium falciparum* (*Pf*) infections. The World Health Organization (WHO) estimated morbidity and mortality due to malaria in 2023 to be 263 million and 597 000, respectively. These are marked increases compared to pre-COVID-19 pandemic numbers in 2019 where 233 million cases and 576 000 deaths were estimated by the WHO. This is in contrast with the steady decline in malaria incidences reported between 2000 and 2014, although they had already started to plateau since 2015.^1^ Several factors have influenced the reversal of the gains previously made to combat malaria. These include a rise in insecticide and drug resistant vectors and parasites, respectively.^2^

The WHO has recently recommended the RTS,S/AS01 and R21/Matrix M malaria vaccines for use in moderate to high transmission endemic areas, and this has led to a decline in childhood mortality due to malaria.^1^ While RTS,S/AS01 achieves only 36% efficacy in infants, R21/Matrix M has ≥75% efficacy, and it is by far the most effective vaccine for seasonal malaria administration.^3,4^ These are still being explored for adult populations and are thus currently limited for full scale use in endemic areas.^5^ Other prevention strategies such as the use of insecticidal bed nets and indoor residual spraying are limited by *Anopheles* resistance to pyrethroids and are thus not effective in eliminating malaria.^6^ Therefore, the mainstay of malaria control relies on chemotherapy, and currently, numerous drugs are used for chemoprophylaxis and treatment of malaria. *Pf* has developed resistance to almost all of these drug classes, with the artemisinins remaining the most effective first-line drugs in clinical use for the treatment of the disease. They are used in combination with other drug classes in Artemisinin-based combination therapy (ACT) regimens.^7^ Recent reports indicating partial artemisinin resistance, as shown by decreased cure rates and high recrudescence following the use of ACT regimens, are alarming. While these were largely noted in Southeast Asia, parasites with novel resistance markers or similar in the *Pf*Kelch13 gene have recently been reported in Africa.^7,8^ This signifies that the parasite has developed tolerance to artemisinins and thus a threat for imminent resistance, compounded by the bleak prospects of novel antimalarials for clinical use in the near future.

Novel antimalarial drugs and chemotypes are urgently needed to combat malaria and overcome current drug resistance challenges. In recent years, the benzoxaborole scaffold has shown promise for drug discovery in various disease areas ranging from parasitic to bacterial infections.^9^ Recent reports of this class with activity against parasites such as *Toxoplasma gondii, Cryptosporidium, Leishmaniasis* and *Pf* are promising for their exploration as a novel chemotype.^8,10,11^ Although the exact target has not been fully deconvoluted, these compounds have been proposed to inhibit the Leucyl-RNA synthetase (LeuRS) by arresting editing by LeuRS or targeting the pre-mRNA processing Cleavage and Polyadenylation Specificity Factor 3 (CPSF3). A number of other proteins including those involved in ubiquitination such as SUMO-activating enzyme subunit 2 and ubiquitin-activating enzyme 1 have also been implicated as targets for the benzoxaboroles.^12–14,11,15^

Recently a preclinical candidate, benzoxaborole **1** (AN13762), was reported with *in vitro* antiplasmodium activity against *Pf*3D7 (IC_50_ = 42 nM) and *Pf*W2 (IC_50_ = 18 nM) (Figure 1). Biochemical and genetic studies of the mechanism of action of this compound revealed that in addition to *Pf*CPSF3, mutations were observed in prodrug activation and resistance esterase (*Pf*PARE) suggesting that the amide of **1** is cleaved to give its carboxylic acid derivative **2** (AN10248, Figure 1). However, the development of AN13762 was reported to have been halted due to toxicity in animals.^15^ Metabolite **2** was roughly equipotent to parent compound **1** against the parasite. The ease of amide hydrolysis to carboxylic acid by the parasite is limiting due to the facile resistance resultant from the *Pf*PARE mutation. AN10248 itself would likely show reduced permeability (and hence bioavailability) *in vivo* and would be susceptible to metabolic processes commonly associated with carboxylic acids.^16^ Hence, there is a need for more stable benzoxaboroles if this promising class is to yield future antimalarials.

**Figure 1:**
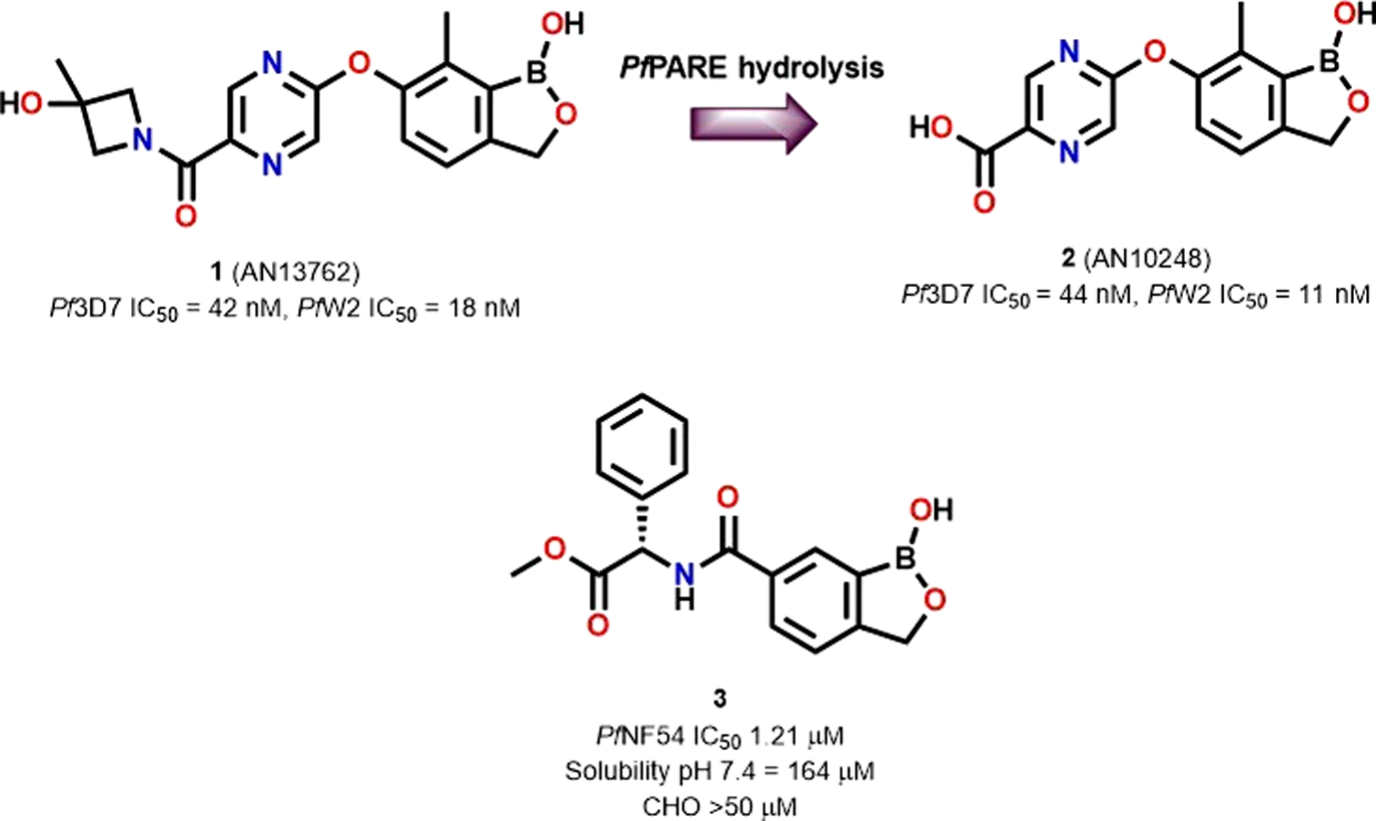
Reported benzoxaborole compounds **1** and **2** and novel phenylglycine-based benzoxaborole hit compound **3**.

Our group recently reported a crystallographic study of boron-containing compounds that inhibited the bacterial Penicillin Binding Protein.^17^ Inspired by the precedence of antimalarial benzoxaboroles mentioned above, we cross-screened these reported compounds for *in vitro* antiplasmodium activity against the drug-sensitive *Pf*NF54 strain and identified hit compound **3** (Figure 1) with an IC_50_ = 1.21 μM and no cytotoxicity against the Chinese Hamster Ovarian cell line (CHO) at the highest concentration tested (IC_50_ > 50 μM). Compound **3** had a high aqueous solubility of 164 μM at pH 7.4 in PBS. Based on these data, we conducted a Formal Hit Assessment (FHA) campaign and explored structure–activity relationship (SAR) studies or variations of the amino acid group appendage of **3** to identify compounds with improved antiplasmodium activity.

Four SAR exploration strategies were undertaken, maintaining the benzoxaborole core unchanged as in **3** (SAR1-SAR4, Figure 2). Starting with commercially sourced carboxylic acid intermediate **5** (Scheme 1), SAR1 focused on the assessment of the stereospecificity of the putative target by synthesizing the opposite enantiomer **6** of **3** and the corresponding racemic mixture **7**. SAR2 explored different substituents on the phenylglycine group including methyl and ethyl esters via compounds **8**–**26** (Table 1). The selection of substituents on the phenylglycine were guided by the Craig plot with groups selected from different quadrants based on their hydrophobicity and electronic nature.^18^ This SAR also incorporated derivatives with the methyl ester of **3** replaced with methylamine and dimethylamine carboxamides affording **27** and **28**, respectively. SAR3 focused on the carboxylic acid matched pairs **29**–**34** of selected methyl and ethyl esters while SAR4 incorporated several discrete modifications that included aromatic carboxamide substituents **35**–**36**, phenylalanine (**37**) and tyrosine (**38**) replacements for the phenylglycine and, a reversed amide **41** (Table 2).

**Figure 2:**
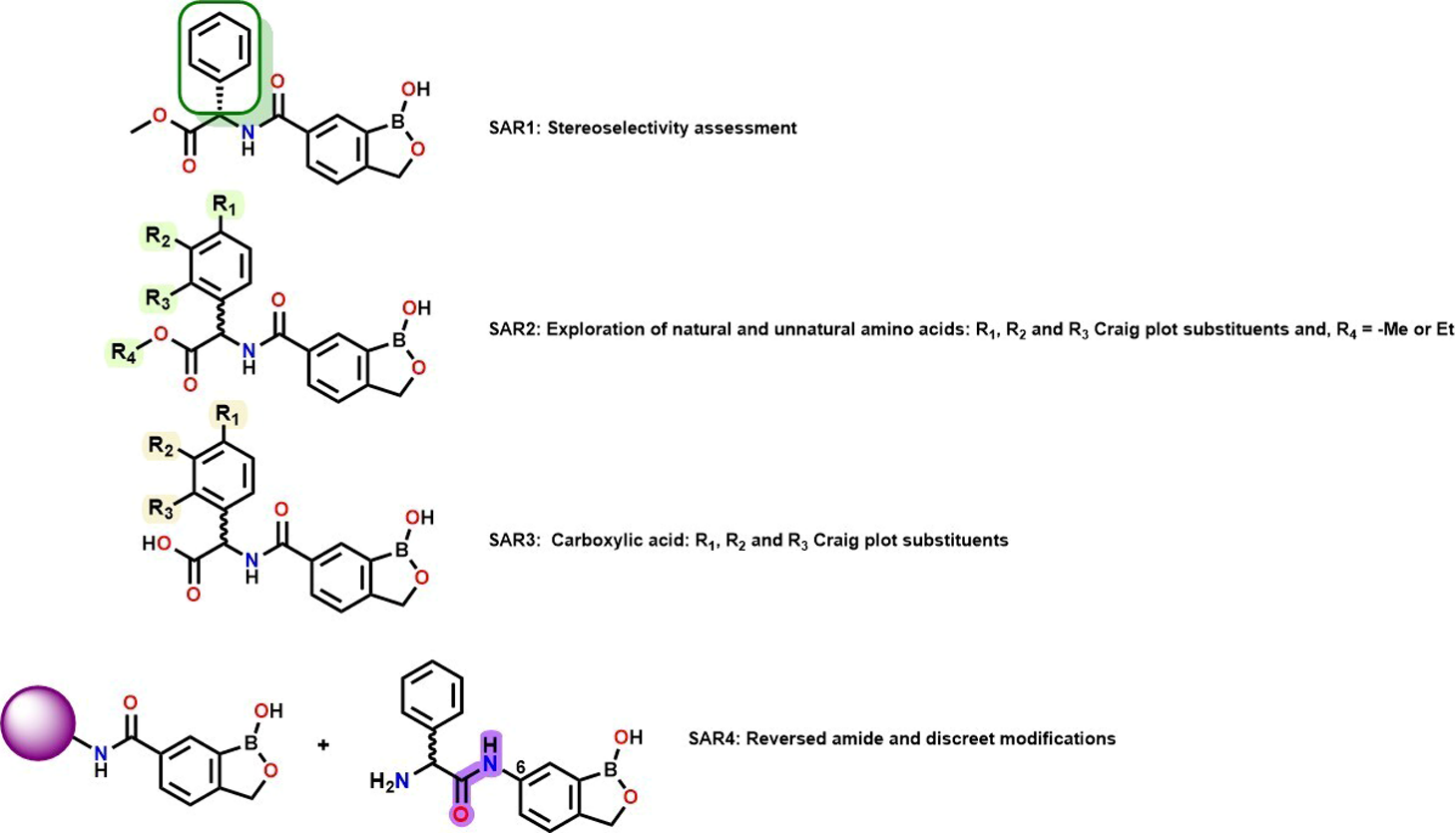
Proposed SAR designs based on **3**.

**Scheme 1:**
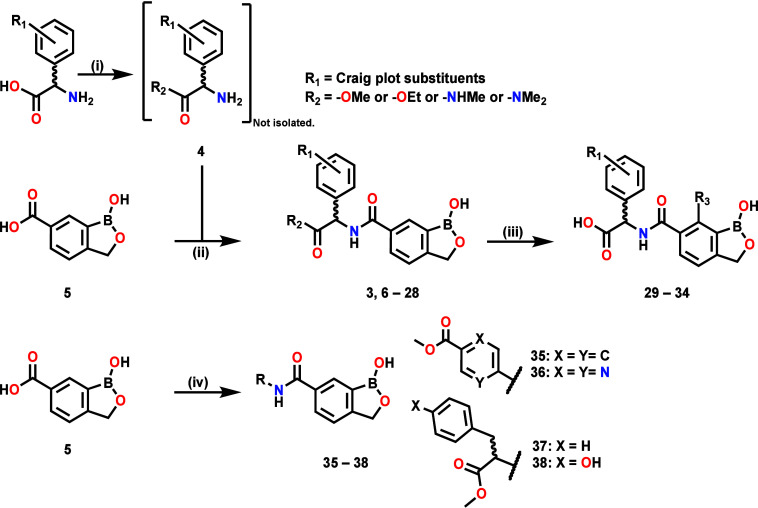
Synthesis of Benzoxaborole Compounds[Fn s1fn1]

* Reagents and conditions: (i) SOCl_2_, MeOH or EtOH, 70 or 80 °C, 16 h; (ii) Relevant ester or amide derivative **4**, EDCI, HOBt, DMF, 27 °C, 14 h or HATU, DIPEA, DMF, 27 °C, 14 h or T3P, Et_3_N, DMF, 27 °C, 14 h; (iii) LiOH·H_2_O, 1,4-dioxane:H_2_O (3:1), 25 °C, 3 h; (iv) = (ii).

**Table 1:**
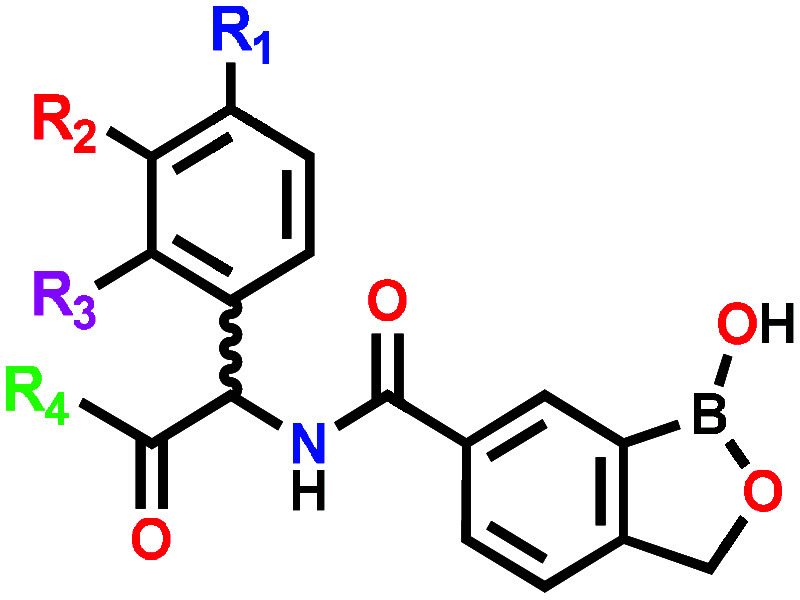
*In Vitro Pf*NF54 and *Pf*K1 Blood Stage Antiplasmodium Activity, Solubility, and Cytotoxicity of SARs 1, 2, and 3 Benzoxaboroles^a^

					^b^ ^,^ ^c^ *Pf*IC_50_ (μM)					
Compound (stereochemistry)	R_1_	R_2_	R_3_	R_4_	NF54	K1	^d^CHO IC_50_ (μM)	^e^SI	^f^RI	^g^Sol. (μM)
Chloroquine					^b^0.007/^c^0.01	^b^0.168/^c^0.1				
3 (*S*)	H	H	H	-OMe	^b^1.21	[Table-fn t1fn8]N.D.	>50			164
6 (*R*)	H	H	H	-OMe	^b^0.64	^b^3.88	>50	>47	6.06	200
7 (*R*,S)	H	H	H	-OMe	^b^0.85	^b^2.67	>50	>59	3.14	150
8	-Me	H	H	-OMe	^b^2.41		>50			90
9	-F	H	H	-OMe	^b^0.72		>50			120
10	H	-F	H	-OMe	^b^1.39		>50			130
11	-OMe	H	H	-OMe	^b^1.19		>50			80
12	H	-OMe	H	-OMe	^b^2.38		>50			75
13	H	H	-OMe	-OMe	^b^>6		>50			125
14	-OH	H	H	-OMe	^b^2.22		>50			120
15	-CF3	H	H	-OMe	^b^2.29		^d^>50			30
16	H	H	-CF3	-OMe	^b^0.93		^d^>50			160
17	-Cl	H	H	-OMe	^b^1.28	^b^2.27	^d^>50		1.8	125
18	H	-Cl	H	-OMe	^b^>6		^d^>50			70
19	H	H	-Cl	-OMe	^b^2.07		^d^>50			140
20	H	H	H	-OEt	^b^1.21		^d^>50			60
21	-Cl	H	H	-OEt	^c^1.38	^c^1.71	N.D.			140
22	H	-Cl	H	-OEt	^c^1.90	^c^2.62	N.D.			80
23	H	H	-Cl	-OEt	^c^3.97	^c^>6	N.D.			40
24	H	H	-Me	-OEt	^c^2.51	^c^2.20	N.D.		0.87	80
25	H	-F	H	-OEt	^c^3.46	^c^4.65	N.D.		1.29	40
26	H	H	-F	-OEt	^c^2.22	^c^2.33	N.D.		1.05	160
27	H	H	H	-NHMe	^b^0.56	^b^0.59	>50		1.05	150
28	H	H	H	-NMe_2_	^b^>6	^b^>6	N.D.			135
29	H	H	H	-OH	^b^0.67		>50	74		150
30	-Me	H	H	-OH	^b^0.12	^b^0.45	>50	>413	3.75	130
31	H	H	-Me	-OH	^b^3.25		N.D.			120
32	H	-Cl	H	-OH	^b^0.39	^b^0.39	*>*50			160
33	H	-F	H	-OH	^b^0.34	^b^0.48	N.D.			160
34	H	H	-F	-OH	^b^1.97	^b^2.77	*>*50			N.D

* ABS antiplasmodium compound activity determination *in vitro* in *P. falciparum*.

a 72 h pLDH.

b 72 h SYBR Green I inhibition assays against drug-sensitive *Pf*NF54 and drug-resistant *Pf*K1 strains. Dose–response curves were generated from at least three independent biological repeats with technical duplicates (mean IC_50_ value *n* = 3 ± S.E.). Chloroquine was used as a reference activity.

c Cytotoxicity evaluated in mammalian CHO cells.

d SI: selectivity index = CHO IC_50_/*Pf*NF54 IC_50_.

e RI: Resistance index *= Pf*K1 IC_50_/*Pf*NF54 IC_50_.

f Sol. = Turbidimetric solubility determined using turbidimetric method in phosphate buffered saline (PBS) at pH 7.4 with hydrocortisone (>200 μM) and reserpine (<10 μM) as controls.

g N.D. = not determined.

**Table 2:**
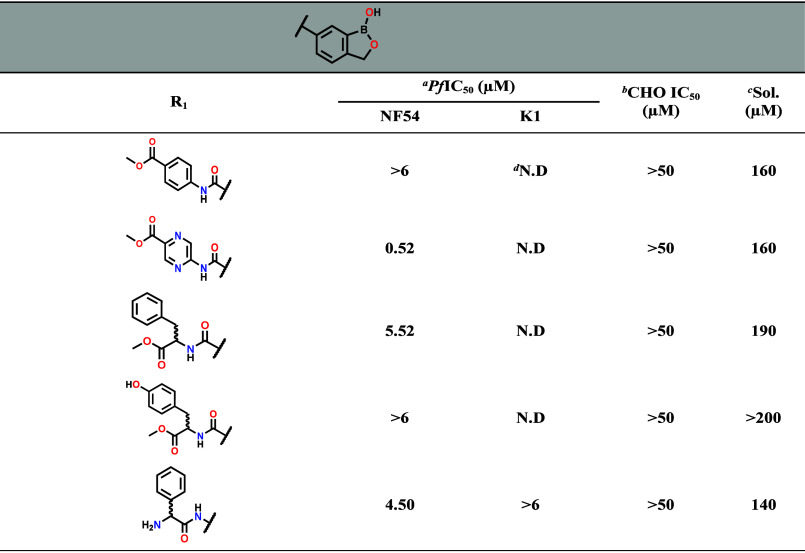
*In Vitro Pf*NF54 and *Pf*K1 Blood Stage Antiplasmodium Activity, Solubility, and Cytotoxicity of SAR 4 Benzoxaboroles^a^

* ABS antiplasmodium compound activity determination *in vitro* in *P. falciparum*.

a 72 h pLDH inhibition assay against drug-sensitive *Pf*NF54 and drug-resistant *Pf*K1 strains. Dose–response curves were generated from at least three independent biological repeats with technical duplicates (mean IC_50_ value *n* = 3 ± S.E.). Chloroquine was used as a reference activity.

b Cytotoxicity evaluated in mammalian CHO cells.

c Sol. = Turbidimetric solubility determined using turbidimetric method in phosphate buffered saline (PBS) at pH 7.4 with hydrocortisone (>200 μM) and reserpine (<10 μM) as controls.

d N.D = not determined.

Synthesis of compounds covered in SAR1–4 (Scheme 1) involved (i) Fischer–Speier esterification of relevant commercially sourced amino acids in methanol or ethanol to give relevant intermediates followed by (ii) and (iv) amide coupling (SAR1, 2, and 4) and (iii) ester hydrolysis (SAR3). The reversed amide compound **41** of SAR4 was synthesized (Scheme 2) via amide coupling of Boc-protected phenylglycine **39** with commercially available 6-amino-benzoxaborole **40** followed by Boc-deprotection under acidic conditions.

**Scheme 2:**
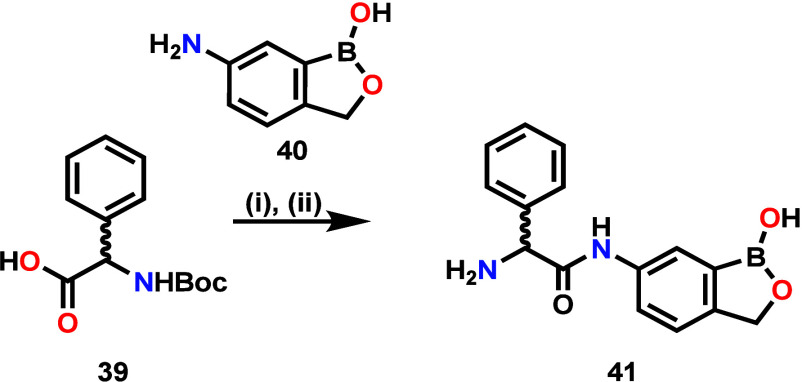
Reversed Amide **53** [Fn s2fn1]

* Reagents and conditions: (i) T3P, Et_3_N, DMF, 27 °C, 14 h; (ii) TFA, DCM, 27 °C, 2 h.

All the compounds were profiled for *in vitro* blood stage antiplasmodium activity against drug-sensitive *Pf*NF54 and multidrug-resistant *Pf*K1 parasites using either the 72 h metabolic pLDH or 72 h proliferative SYBR Green I assays, both of which are known to produce correlative IC_50_ values.^19^ Mammalian cell cytotoxicity was determined using the CHO cell line (Table 1). Hit validation was conducted with resynthesized compound **3**, and its antiplasmodium activity against *Pf*NF54 was confirmed with an IC_50_ of 1.06 μM, comparable to 1.21 μM obtained in the initial screen. We then synthesized both opposite enantiomer **6** starting with the *R*-enantiomer phenylglycine and racemic mixture **7**. The latter was made to assess if the racemate retains sufficient antiplasmodium activity so that the other compounds could be synthesized in racemic form, thereby streamlining synthetic efforts. The *Pf*NF54 IC_50_ values for **6** and the racemic mixture **7** were 0.64 and 0.85 μM, respectively (Table 1). These data show that the opposite enantiomer **6** is 2-fold more active compared to **3** and, that the racemate **7** retains antiplasmodium activity comparable to that of **6**. Given that the racemic mixture had viable blood stage antiplasmodium activity, it became expedient to synthesize all subsequent compounds as racemic mixtures (Scheme 1 and 2).

In SAR2, the most active compounds in the series were *para*-fluoro **9** (*Pf*NF54 IC_50_ = 0.72 μM), and methyl amide **27** (*Pf*NF54 IC_50_ = 0.56 μM) with blood stage antiplasmodium activities comparable to that of **7** (*Pf*NF54 IC_50_ = 0.85 μM), within a 2-fold variation inherent in the assay. The regioisomer of **9**, *meta*-fluoro **10** had slightly lower blood stage antiplasmodium activity against *Pf*NF54 (IC_50_ = 1.39 μM). The dimethyl amide **28** lost blood stage antiplasmodium activity (*Pf*NF54 IC_50_ > 6 μM) at the highest concentration tested. Unlike **6** that showed lower blood stage activity against the drug-resistant *Pf*K1 strain (*Pf*K1 IC_50_ = 3.88 μM), **27** showed equipotent blood stage activity against the *Pf*K1 strain with an IC_50_ = 0.60 μM. Compound **27** also maintained favorable solubility (150 μM) and showed no cytotoxicity at the highest concentration tested (CHO IC_50_ > 50 μM) (Table 1). Other substituents on the phenylglycine in the series led to lower blood stage activity relative to **7**, i.e. *Pf*NF54 IC_50_ > 1 μM. The ethyl ester derivatives **20**–**26** all had lower blood stage antiplasmodium activity with *Pf*NF54 IC_50_ > 1 μM compared to **7** irrespective of the substituent on the phenylglycine. When phenylalanine in **37** and tyrosine in **38** were introduced to explore effects of the spacer and addition of the polar group (OH), respectively, the former was 10-fold less active (*Pf*NF54 IC_50_ = 5.52 μM) while the latter was not active at the highest concentration tested (*Pf*NF54 IC_50_ > 6 μM) (Table 2). Derivatives with the methyl 4-aminobenzoate appendage **35** and aminopyrazine-2-carboxylate appendage **36** had *Pf*NF54 IC_50_ values of >6 and 0.52 μM, respectively. The high blood stage antiplasmodium activity for **36** is of interest in that it contains the pyrazine-2-carboxy moiety present in the preclinical candidate **1** (Figure 1) suggesting that this group may be contributing to the slight increase in potency.

SAR3 with carboxylic acid matched pairs showed the highest *in vitro* blood stage antiplasmodium activity compared to other series with activity ranging between IC_50_ = 0.12–3.25 μM against *Pf*NF54 (Table 1). Compound **30** with the *para*-methyl substituent showed the highest activity (*Pf*NF54 IC_50_ = 0.12 μM) notably more active than **6** and **7**. Against the drug-resistant strains *Pf*K1 and *Pf*Dd2, **30** was slightly less active with IC_50_ values 0.45 and 0.97 μM, respectively. In this series, compounds **32** with *meta*-chloro and **33** with *meta*-fluoro also had the highest combination of blood stage antiplasmodium activity against both *Pf*NF54 (0.39 and 0.34 μM, respectively) and *Pf*K1 (0.39 and 0.48 μM, respectively). When these compounds were evaluated for their transmission blocking activity against immature (iGc > 90% stage II–III) and late stage (lGc > 90% IV–V) gametocytes using a luciferase assay platform, **32** was inactive; however, **33** showed moderate activities against both stages (IC_50_ = 3.95 μM and 4.46 μM against iGc and lGc, respectively). Cytotoxicity evaluation for all the compounds across series in the study shows that regardless of the different antiplasmodium potencies, all the compounds profiled for cytotoxicity in the CHO cell line had an IC_50_ > 50 μM as well as favorable solubility, 80 to >200 μM (Table 1 and 2). The reversed amide **41** (Table 2) led to decreased blood stage activity (*Pf*NF54 IC_50_ = 4.5 μM) but maintained the favorable CHO cytotoxicity (IC_50_ > 50 μM) and solubility (140 μM). Compounds **6** and **30** were evaluated for their hemolytic potential, and no hemolysis was noted across the tested concentration range, indicating that the observed activity is not attributable to host cell damage.

The metabolic stability of selected compounds was then assessed via incubation with human, mouse, and rat liver microsomes for 0.5 h. Compounds **3**, **6**, **9**, **29**, and **30** were metabolically stable across the species with apparent intrinsic clearances (CLint, app) < 11.6 μL/min/mg, predicting low hepatic metabolic clearance *in vivo* (Table 3).

**Table 3:**
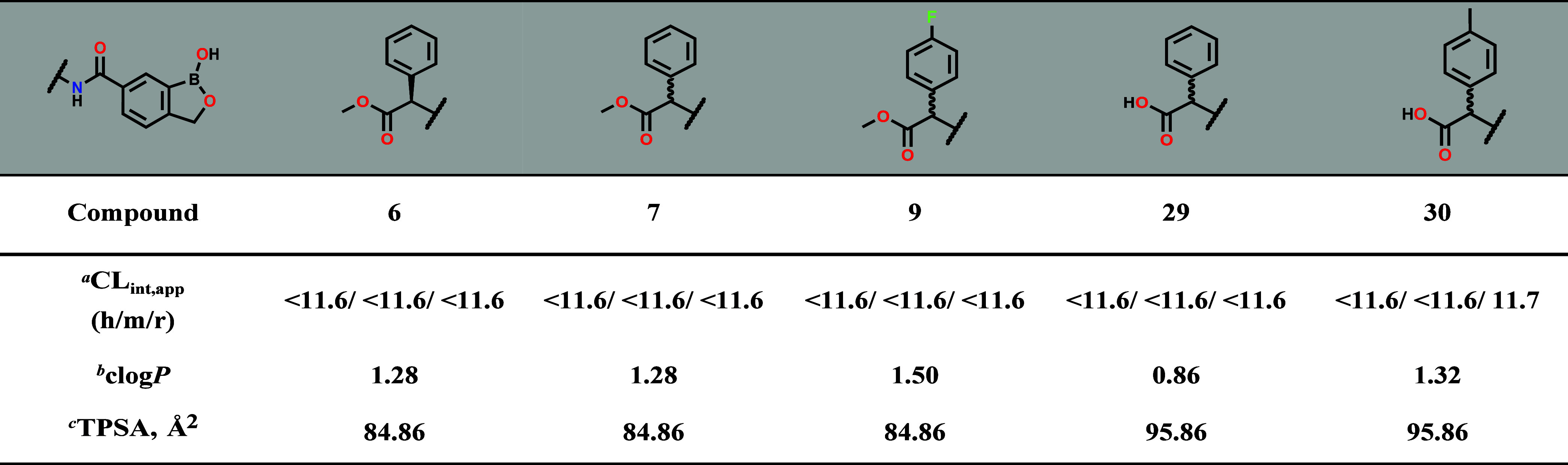
*In Vitro* Microsomal Metabolic Stability of Selected Compounds

a Intrinsic clearance in μL/min/mg.

b clogP = calculated logP.

c TPSA = Total Polar Surface Area, both determined with Stardrop v6.3.

Toward shedding light on the potential novelty of the mechanism of action (MoA) and/or putative targets of these compounds, selected compounds were subjected to targeted hydrophilic metabolomics and barcoded mutant cross-resistant studies.^20,21^ The former was aimed at identifying the pathways in which these benzoxaboroles are acting and the latter to show cross resistance (or lack thereof) with known targets of this class. An assessment of the metabolic fingerprints (metaprints)^20,22^ of compounds **6** and **9** showed a weak increase in pyrimidine biosynthesis precursors (Figure 3). Additionally, compound **6** resulted in a slight increase in hemoglobin-derived peptides and folate intermediates were also observed for compound **7**. However, overall, the metaprint of the compounds studied results in ambiguous profiles under the conditions tested. As previous studies have implicated CPSF3, which is involved in pre-mRNA processing, as a target of benzoxaboroles, it is likely that the pathways perturbed by these compounds do not result in easily defined metabolite changes. Thus, these ambiguous profiles may result from undetectable cellular perturbations as has been previously observed for some compound classes such as trioxolanes and translation inhibitors which give weak signals and, cluster in unclassified pathways.^20^

**Figure 3:**
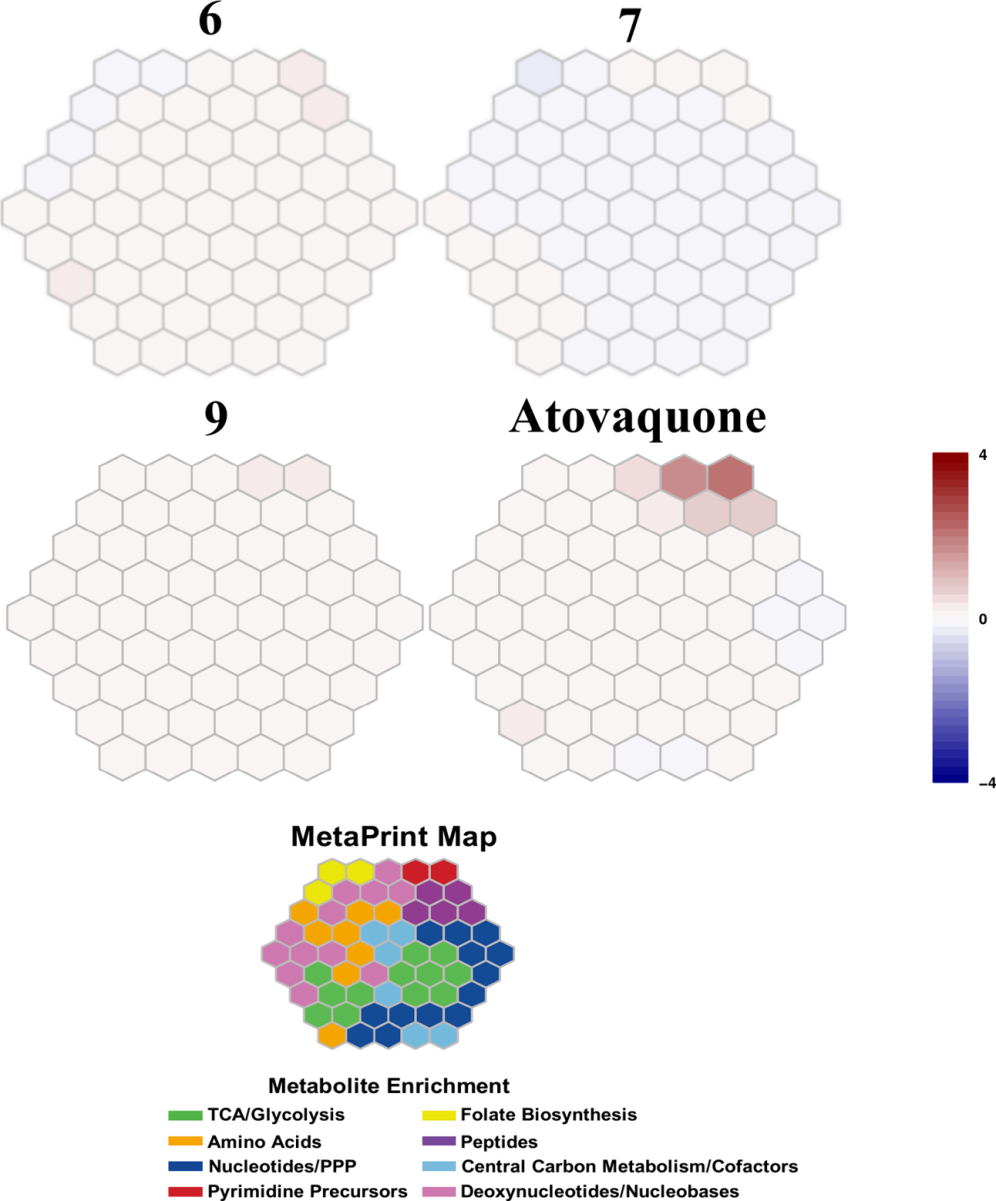
Metabolic fingerprint (MetaPrint) maps of selected benzoxaboroles. Hexagons represent 113 targeted metabolites in metabolite clusters, separated into eight metabolic pathways.^20,22^

Compound **7** was further assessed for cross resistance against a pool of parasite barcoded drug-resistant mutants (Table S2). This compound showed no significant changes in the count of barcode proportions in treated (3 × IC_50_) and untreated parasites in both *Pf*3D7 and Dd2 backgrounds (Figure 4). The data suggest that the compound has no cross-resistance with any of the mutants present in the parasite pool and implies that it is likely acting through a novel MoA. The pool included parasites with the CPSF3-Y408S mutation, previously reported in resistant parasite lines raised against **AN3661**, and compound **7** was not cross-resistant to these parasite lines.^14^ This further suggested that this new class of benzoxaboroles is acting through a novel MoA, dissimilar to other benzoxaborole classes, and that it has no cross-resistance to the current antimalarials.

**Figure 4:**
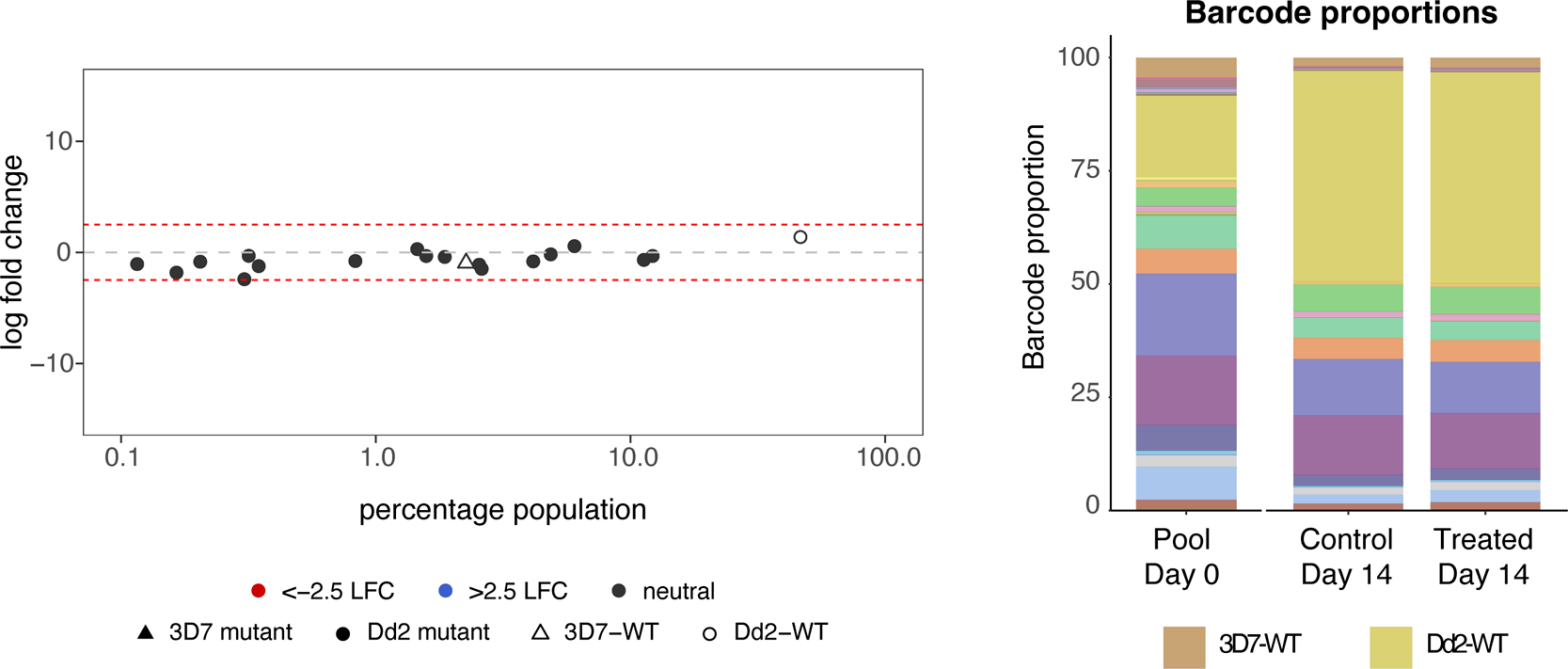
Profile of **7** against an *in vitro* library of barcoded drug-resistant *Pf*3D7 and *Pf*Dd2 lines. (*Left panel*) Log2 fold-change of each line after treatment with **7** relative to starting abundance, with each dot representing a specific mutant. (*Right panel*) Barcode proportions of each line in the pool at day 0, and at day 14 for the untreated control or exposed to 3 × IC_50_ of **7**.

As the ester groups are metabolically labile being prone to hydrolysis, and that *Pf*PARE has been reported to hydrolyze pepstatin-based antimalarials, future work should include selection of resistant mutants *in vitro* to determine if this class of benzoxaboroles also shows *Pf*PARE resistant markers.^23^ It is indeed possible that the observed superior activity of the methyl esters compared to the ethyl esters (Table 1) may be due to the former being hydrolyzed faster by *Pf*PARE or other esterases.

Given the promising *in vitro* metabolic stability and blood stage antiplasmodium data, compounds **6**, **7**, **9**, **30**, **32**, and **33** were evaluated for *in vivo* efficacy in the *P. berghei* mouse infection model. When dosed orally in a 50 mg·kg^–1^ standard quadrupole dosing regimen, compounds **6** and **7** showed 74 and 78% reduction in parasitaemia, respectively, with mean survival days of 7 days (Table 4). Pharmacokinetic evaluation of **6** in healthy Balb/c mice shows that the compound has a low clearance (20 mL·min^–1^·kg^–1^) and a high volume of distribution (*V*_d_ = 34.4 L·kg^–1^), which results in a long half-life (Table 5 and Figure 5). This, combined with good oral exposure (bioavailability = 43%) would have supported the observation of good efficacy for compound **6**. However, when the dose of the compound was increased to 50 mg/kg, the increase in exposure was less than dose proportional, suggesting saturation of absorption. The lower-than-expected exposures would not maintain coverage above the IC_50_ for the 24 h period between doses, which explains why **6** was not more efficacious *in vivo*.

**Table 4:**
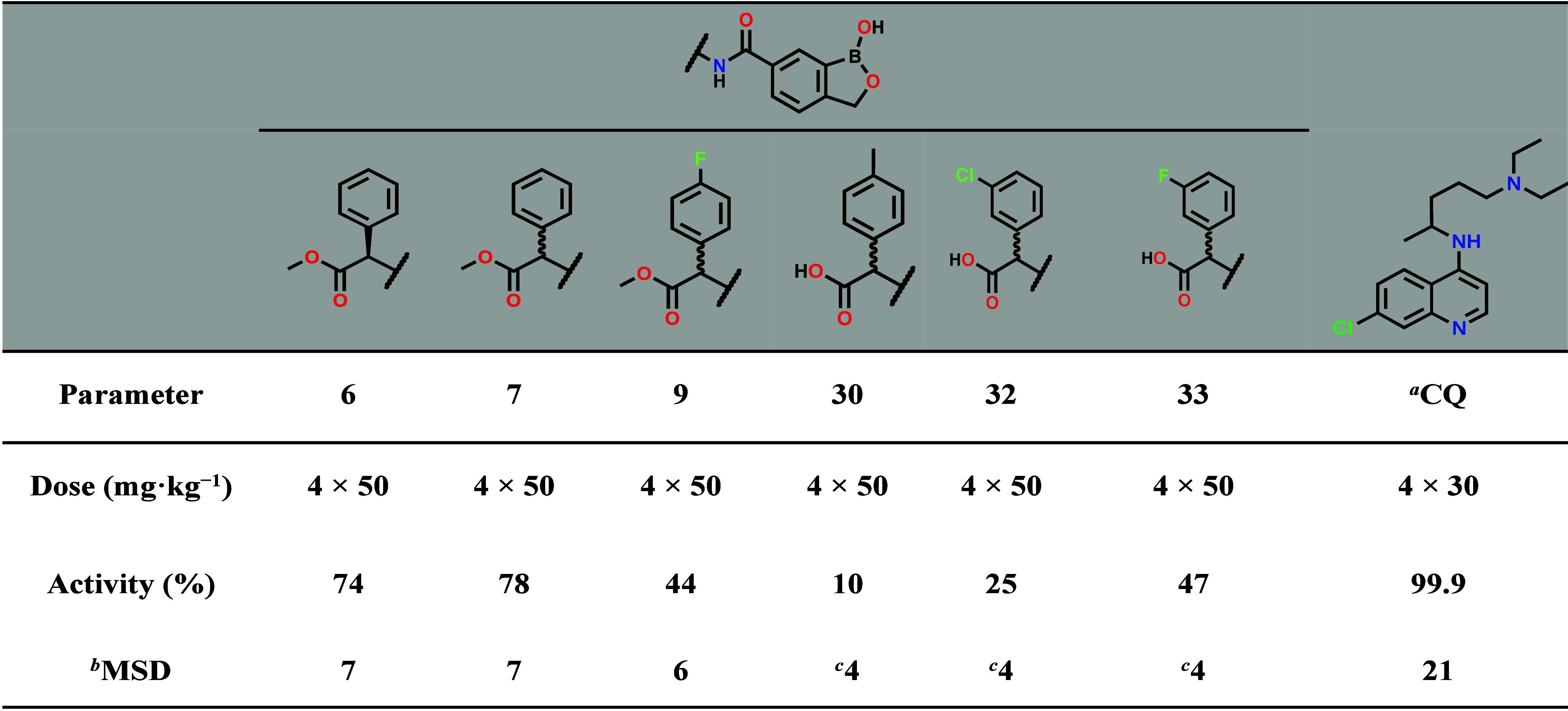
*In Vivo* Efficacy of Selected Benzoxaboroles Following Oral Dosing in *P. berghei* Malaria Infection Mice Model

a CQ = chloroquine.

b MSD = mean survival days.

c Mice were euthanized on day 4.

**Table 5:**
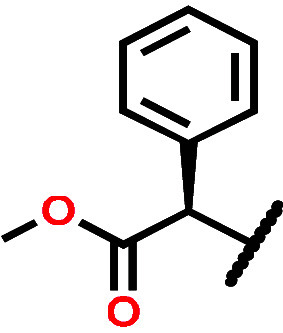
Mouse Pharmacokinetic Parameters of 6

	6	
Parameter	iv	oral
Dose (mg·kg^–1^)	3.0	10
*C*_max_ (μM)	-	3.5
*T*_max_ (h)	-	0.5
AUC (μM·min^–1^)/(min·μmol/L)	307	508
*V*_d_ (L·kg^–1^)	34.4	-
CL_B_ (mL·min^–1^·kg^–1^)	20.0	-
apparent *t*_1/2_ (h)	21.9	70.5
*F* (%)	-	43.4

**Figure 5:**
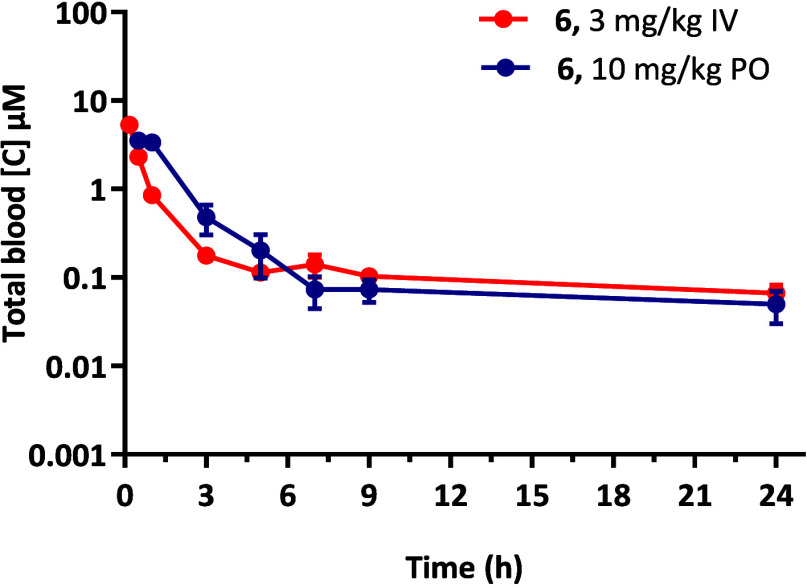
Blood concentrations of compound **6** following intravenous (IV) and oral (PO) dosing in healthy BalbC mice.

Compounds **9**, **30**, **32**, and **33** showed lower efficacy *in vivo*, 10–47% reduction in parasitaemia (Table 4). The suboptimal efficacy of the carboxylic acid derivatives compared to the ester compounds contrasts with *in vitro* blood stage antiplasmodium data, which shows better activity of the former compared to the latter. This may be due to low permeability of the carboxylic acid derivatives on oral dosing resulting from low diffusion across intestinal epithelial cells and thus, lower drug exposure.^16^

Benzoxaboroles are emerging as a promising scaffold for development of next generation antimalarials with advantages including activity likely against novel target(s), drug-like properties, and selectivity for the parasite over mammalian cells. The FHA campaign herein enclosed reports on the promising hit compounds with high cross species *in vitro* microsomal metabolic stability, and SAR exploration identified submicromolar hit compounds with potential for further optimization into lead compounds. Compound **30** showed the highest blood-stage antiplasmodium activity (*Pf*NF54 IC_50_ = 0.12 μM) *in vitro*. Other carboxylic acid derivatives, **32** and **33** also showed the high blood-stage antiplasmodium activity in *in vitro* assays adding to the report of the carboxylic acid derivative **2** having activity against *Pf* similar to that of amide **1** (Figure 1). That ester derivatives had efficacy *in vivo* may be due to the proclivity of the esters to undergo metabolic hydrolysis to active carboxylic acids, suggesting that these compounds likely act as prodrugs *in vivo*. Lack of cross-resistance and ambiguous metabolomics profile of selected benzoxaboroles suggest a novel MoA for this class. Further biochemical studies are required around the esters and amides of the current series to ascertain *Pf*PARE’s involvement for resistance development and, for prodrug convertase activity as well as the relevance to antiplasmodium activity of the corresponding acids. Given inconclusive knowledge of the exact target of the benzoxaboroles and observed polypharmacology of this compound class,^24^ additional studies will be essential to identify target/s for these compounds. The pharmacokinetic data of compound **6** with respect to moderate oral bioavailability and long half-life are promising for lead optimization for an oral drug. This FHA campaign has identified a new class of benzoxaborole compounds with *in vivo* efficacy, which motivates further optimization for potential hit-to-lead transition.

## Supplementary Material



## Notes

All intermediates and target compounds were characterized using NMR, and purity was determined using LC-MS; experimental procedures, characterization of key intermediates as well as final compounds, description of biochemical, solubility, and metabolic stability assays are provided. *In vitro* gametocytocidal activity of selected compounds is included (PDF)

## □

Mokhitli Morake – Centre for Targeted Protein Degradation (CeTPD), University of Dundee, 1 James Lindsay Place, Dundee, DD1 5JJ, United Kingdom

## Notes

A portion of content of this manuscript has previously appeared in the Ph.D. thesis of M.M.^25^

## Abbreviations

ACT: Artemisinin-based Combination Therapy
CPSF3: Cleavage and Polyadenylation Specificity Factor 3
FHA: Formal hit assessment
LeuRS: Leucyl-RNA synthetase
*Pf*: *Plasmodium falciparum*
WHO: World Health Organization
