# Supplementary material for: A Novel Class of Orally Bioavailable Phenylglycine–Benzoxaborole Conjugates with Antimalarial Activity and Potentially Novel Mechanism of Action
Source: ACS Med Chem Lett. 2025 Dec 11;17(1):22–31. doi: 10.1021/acsmedchemlett.5c00549 (PMC12794096; doi:10.1021/acsmedchemlett.5c00549)
Supplement: Supplementary file 1 [file ml5c00549_si_001.pdf]

A novel class of orally bioavailable phenylglycine-benzoxaborole conjugates with antimalarial activity and potentially novel mechanism of action

Mokhitli Morake,[†] Dale Taylor,[§] Dina Coertzen,[Ω] Mathew Njoroge,[§] Liezl Krugmann,[§] Meta Leshabane,[Ω] Shanté da Rocha,[Ω] Tarrick Qahash,[Ψ,Φ] Gareth Girling,[♦] Rachael Coyle,[♦,Δ] Marcus C. S. Lee,[♦,Δ] Sergio Wittlin,[‖,⊥] Manuel Llinás,[Ψ,Θ,Φ] Lyn-Marie Birkholtz,[Ω,†] Gregory S. Basarab,[φ] and Kelly Chibale[†,§,Π,‡,*]

[†]Department of Chemistry, University of Cape Town, Rondebosch 7701, South Africa.

[§]Drug Discovery and Development Centre (H3D), Institute of Infectious Disease and Molecular Medicine (IDM), University of Cape Town, Observatory, 7925, South Africa.

[Ω]Department of Biochemistry, Genetics & Microbiology, Institute for Sustainable Malaria Control, University of Pretoria, Private Bag X20, Hatfield 0028, Pretoria, South Africa.

[Ψ]Department of Biochemistry and Molecular Biology, The Pennsylvania State University, State College, PA 16802, United States of America.

[Φ]Huck Center for Malaria Research, The Pennsylvania State University, State College, PA 16802, United States of America.

[♦]Wellcome Sanger Institute, Wellcome Genome Campus, Hinxton, United Kingdom.

[Δ]Biological Chemistry and Drug Discovery, School of Life Sciences, University of Dundee, Dundee, United Kingdom.

[‖]Swiss Tropical and Public Health Institute, Socinstrasse 57, 4002 Basel, Switzerland.

[⊥]University of Basel, 4003 Basel, Switzerland.

[Θ]Department of Chemistry, The Pennsylvania State University, State College, PA 16802, United States of America.

[†]Department of Biochemistry, Stellenbosch University, Stellenbosch, 7600, South Africa

[φ]Drug Discovery and Development Centre (H3D), Department of Chemistry, University of Cape Town, Rondebosch 7701, South Africa.

[Π]South African Medical Research Council Drug Discovery and Development Research Unit, University of Cape Town, Rondebosch 7701, South Africa

[‡]Institute of Infectious Disease and Molecular Medicine, University of Cape Town, Rondebosch 7701, South Africa.

Table of Contents

Compounds Synthesis and characterization 3

Synthesis of the methyl and amides 3

Selected methyl esters 4

Selected amides 5

Synthesis of carboxylic acid derivatives 5

Selected carboxylic acids 6

Aqueous solubility 7

In vitro antiplasmodium Assay 7

The endpoint parasite lactate dehydrogenase assay 7

The SYBR Green I assay 8

In vitro activity against gametocyte stage 8

In vitro cytotoxicity Assays 9

In vitro cytotoxicity evaluation against Chinese Hamster Ovarian (CHO) cell line 9

In vitro hemolytic evaluation in red blood cells 10

In vitro Metabolic Stability Assay 10

In vivo Antiplasmodium Assay (Swiss TPH) 10

In vivo Pharmacokinetic Studies in Healthy Mice (H3D, DMPK) 11

Barcoded Cross-resistance Studies 12

Metabolomics 14

Spectra data of key compounds 15

References 23

Compounds Synthesis and characterization

All commercially available chemicals were purchased from Sigma-Aldrich (South Africa and Germany), AK Scientific (United States), Fluorochem (United Kingdom) or Combi-Blocks Limited (United States) and were of analytical grade and thus used without further purification. Reaction progress was monitored via analytical thin layer chromatography (TLC) sourced from Merck (South Africa) as TLC Silica gel 60 F_{254} aluminium-backed plates and were visualized under ultraviolet light lamp at 254 nm. Compounds were purified on silica gel chromatography using Merck kieselgel 60 (70 – 230 mesh) by gravity column chromatography or flash column chromatography on a Biotage Isolera™ system (Biotage AB, Uppsala, Sweden). ^{1}H NMR (for all intermediates and final compounds) and ^{13}C NMR (i.e. for target compounds only) were recorded on Varian Mercury (^{1}H 300 MHz) and Bruker Ascend™ 600 (^{1}H 600, ^{13}C 151 MHz). Chemical shifts (δ) are reported in parts per million (ppm) downfield trimethylsilane as an internal standard and coupling constants (*J*) are reported in Hertz (Hz). Purity and molecular ions of target compounds were acquired on an Agilent HPLC system equipped with Agilent 1260® Infinity Binary Pump, Agilent 1260® Infinity Diode Array Detector, Agilent 1290® Infinity Column Compartment, Agilent 1260® Infinity Autosampler, Agilent 6120® Quadrupole mass spectrometer with APCI and ESI multimode ionization source. Target compounds tested for biological activity were confirmed to have ≥ 95% purity.

Synthesis of the methyl and amides

Method A: To a solution of 1-hydroxy-1,3-dihydrobenzo[c][1,2]oxaborole-6-carboxylic acid (1 mmol, 1 eq.), EDCI (0.15 mmol, 1.5 eq.) and HOBt (0.15 mmol, 1.5 eq.) in DMF (1 mL) was added DIPEA (2 mmol, 1.0 eq.) and reaction mixture was stirred for 30 min at 0 °C. To this solution amine (0.13 mmol, 1.3 eq.) was added and the reaction stirred at 20 °C for 14 h. *Method B*: To a solution of 1-hydroxy-1,3-dihydrobenzo[c][1,2]oxaborole-6-carboxylic acid (1 mmol, 1.0 eq.) and HATU (1.5 mmol, 1.5 eq.) in DMF (2 mL) was added DIPEA (2 mmol, 2.0 eq.). The mixture was stirred at 24 °C for 30 min, and then amine (1.5 mmol, 1.5 eq.) was added. The resulting mixture was stirred at 24 °C for 14 h. *Method C*: To a solution of 1-hydroxy-1,3-dihydrobenzo[c][1,2]oxaborole-6-carboxylic acid (1 mmol, 1.0 eq.), amine (1.5 mmol, 1.5 eq.) and triethylamine (2 mmol, 2.0 eq.) in DMF (2 mL) was added T3P® (1.5 mmol, 1.5 eq.). The mixture was stirred at 24 °C for 14 h.

Work-up (*Method A, B and C*): At the end of the reaction time, reaction mixture was diluted with EtOAc (2 mL) and then toluene (3 x 5 mL) while concentrating it under reduced pressure. The residue was taken up into EtOAc (5 mL) and a saturated $NaHCO_3$ solution was added (5 mL) followed by extraction in EtOAc (3 x 5 mL). The organic layer was washed with brine (10 mL), dried on

Na_2SO_4, and concentrated under reduced pressure. The resulting residue was adsorbed on isolute and submitted to reverse-phased Biotage (for final compounds only) eluting with MeOH (0 – 100%) in water to afford the desired amides **3 - 28** and **35 - 41**.

Selected methyl esters

***Methyl (S)-2-(1-hydroxy-1,3-dihydrobenzo[c][1,2]oxaborole-6-carboxamido)-2- phenylacetate,* 3**

Obtained through method A as a white solid (57 mg, 16%). m.p: 142-143 °C; R_f (EtOAc:Hex:EtOH, 3:6:1) 0.15; ^{1}H NMR (600 MHz, MeOD) δ 8.15 (s, 1H), 7.95 (d, J = 8.0 Hz, 1H), 7.50 – 7.45 (m, 3H), 7.43 – 7.32 (m, 3H), 5.72 (s, 1H), 5.12 (s, 2H), 3.76 (s, 3H). ^{13}C NMR (151 MHz, MeOD) δ 172.8, 170.3, 158.9, 137.5, 134.5, 131.3, 130.6, 129.9 (2C), 129.6, 129.1 (2C), 122.5 (2C), 72.3, 58.9, 53.1. LC-MS (APCI$^+$/ESI): m/z = 326.1 $[M+H]^+$, calculated exact mass for $C_{17}H_{16}BNO_5$: 325.11. Purity > 99%, t_R = 2.309 min.

***Methyl (R)-2-(1-hydroxy-1,3-dihydrobenzo[c][1,2]oxaborole-6-carboxamido)-2- phenylacetate,* 6**

Obtained through method B a white solid (52 mg, 28%). m.p: 130-132°C; R_f (EtOAc:Hex:EtOH, 3:6:1) 0.15; ^{1}H NMR (600 MHz, MeOD) δ 8.15 (s, 1H), 7.95 (d, J = 8.0 Hz, 1H), 7.49 – 7.46 (m, 3H), 7.41 – 7.34 (m, 3H), 5.72 (s, 1H), 5.12 (s, 2H), 3.75 (s, 3H). ^{13}C NMR (151 MHz, MeOD) δ 172.8, 170.2, 158.9, 137.5, 134.2, 131.4, 130.7, 129.9, 129.6, 129.1, 122.5 (2C), 72.3, 58.9, 53.1. LC-MS (APCI$^+$/ESI): m/z = 326.1 $[M+H]^+$, calculated exact mass for $C_{17}H_{16}BNO_5$: 325.11. Purity: 96%, t_R = 2.321 min.

***Methyl 2-(1-hydroxy-1,3-dihydrobenzo[c][1,2]oxaborole-6-carboxamido)-2-phenylacetate,* 7**

Obtained through method A as a white solid (37 mg, 10%). m.p: 178-181°C; *Rf* (DCM:MeOH, 9:1) 0.2; ^{1}H NMR (400 MHz, MeOD) δ 8.14 (s, 1H), 7.94 (d, J = 8.0 Hz, 1H), 7.50 – 7.32 (m, 5H), 5.73 (s, 1H), 5.11 (s, 2H), 3.75 (s, 3H). ^{13}C NMR (101 MHz, MeOD) δ 171.4, 168.9, 157.4, 136.1, 132.7, 129.8 (2C), 129.2 (2C), 128.5, 128.2, 127.6, 121.0 (2C), 70.8, 57.5, 51.7. LC-MS (APCI$^+$/ESI): Purity > 99%, t_R = 2.309 min, m/z = 326. $[M+H]^+$, calculated exact mass for $C_{17}H_{16}BNO_5$: 325.11.

Selected amides

1-hydroxy-N-(2-(methylamino)-2-oxo-1-phenylethyl)-1,3-dihydrobenzo[c][1,2]oxaborole-6-carboxamide, **27**

Obtained through method A as a beige solid (15 mg, 8.23%). m.p: 216-218°C; R_f (EtOAc: Hex: EtOH, 3:6:1) 0.6; ^{1}H NMR (400 MHz, MeOD) δ 8.16 (s, 1H), 7.96 (d, J = 8.0 Hz, 1H), 7.52 - 7.46 (m, 3H), 7.41 – 7.31 (m, 3H), 5.64 (s, 1H), 5.12 (s, 2H), 2.77 (s, 3H). ^{13}C NMR (101 MHz, MeOD) δ 173.1, 169.8, 158.9, 139.0, 134.2, 131.2, 130.5, 129.8 (2C), 129.3, 128.8 (2C), 122.5 (2C), 72.2, 59.3, 26.5. LC-MS (APCI$^+$/ESI): *m/z* = 325.1 [M+H]$^+$, calculated exact mass for $C_{17}H_{17}BN_2O_5$: 324.14. Purity >99%, t_R = 2.317 min.

N-(2-(dimethylamino)-2-oxo-1-phenylethyl)-1-hydroxy-1,3-dihydrobenzo[c][1,2]oxaborole-6-carboxamide, **28**

Obtained through method A as a beige solid (12 mg, 22%). m.p: 179-181°C; R_f (DCM:MeOH, 9:1) 0.2; ^{1}H NMR (600 MHz, MeOD) δ 8.13 (s, 1H), 7.94 (d, *J* = 8.0 Hz, 1H), 7.47 – 7.43 (m, 3H), 7.40 – 7.34 (m, 3H), 6.09 (s, 1H), 5.10 (s, 2H), 3.01 (s, 6H). ^{13}C NMR (151 MHz, MeOD) δ 171.9, 169.4, 158.9, 137.8, 134.3, 131.2, 130.5, 130.1 (2C), 129.6, 129.4 (2C), 122.5 (2C), 72.2, 56.3, 37.4, 36.4. LC-MS (APCI$^+$/ESI): *m/z* = 339.1 [M+H]$^+$, calculated exact mass for $C_{18}H_{19}BN_2O_4$: 338.14. Purity >99%, t_R = 2.295 min.

2-amino-N-(1-hydroxy-1,3-dihydrobenzo[c][1,2]oxaborol-6-yl)-2- phenylacetamide, **41**

Obtained through method C as an orange solid (120 mg, 63% over two steps). m.p: 196-198°C; R_f (DCM:MeOH, 9:1) 0.1; ^{1}H NMR (600 MHz, MeOD) δ 7.79 (s, 1H), 7.62 (d, *J* = 8.3, 1H), 7.57 (d, *J* = 7.5 Hz, 2H), 7.48 - 7.41 (m, 3H), 7.31 (d, *J* = 8.3 Hz, 1H), 5.02 (s, 2H), 4.95 (s, 1H). ^{13}C NMR (151 MHz, MeOD) δ 168.9, 151.3, 138.2, 136.5, 130.6 (2C), 130.4 (2C), 128.9 (2C), 124.2, 122.6 (2C), 72.0, 59.1. LC-MS (APCI$^+$/ESI): *m/z* = 283.1 [M+H]$^+$, calculated exact mass for $C_{15}H_{15}BN_2O_3$: 282.12. Purity > 99%, t_R = 1.206 min.

Synthesis of carboxylic acid derivatives

To a solution of an appropriate ester (1 mmol, 1 eq.) in dioxane:H_2O (3:1) was added LiOH·H_2O (2.5 mmol, 2.5 eq.) at 24 °C. The reaction mixture was left to stir for 12 h and to this solution, 1 N HCl was added dropwise to bring solution to pH = 5. The resulting solution was extracted with EtOAc (3

x 5 mL) and the combined organic layer was washed with brine (10 mL), dried on Na_2SO_4, and concentrated under reduced pressure. The resulting residue was adsorbed on isolute and submitted to reverse-phased Biotage eluting with MeOH (0 – 100%) in water. Fractions containing the product were combined and concentrated *in vacuo* to afford desired carboxylic acids **29** – **34**.

Selected carboxylic acids

Methyl(R)-2-(1-hydroxy-1,3-dihydrobenzo[c][1,2]oxaborole-6-carboxamido)-2-phenylacetate, **29**

Obtained as a white solid (29 mg, 43%). m.p: 154-157°C; ^{1}H NMR (400 MHz, MeOD) δ 8.13 (s, 1H), 7.94 (d, *J* = 8.0 Hz, 1H), 7.50 – 7.32 (m, 5H), 5.73 (s, 1H), 5.12 (s, 2H). ^{13}C NMR (101 MHz, MeOD) δ 171.4, 168.9, 157.4, 136.1, 132.7, 129.8, 129.2 (2C), 128.5, 128.2, 127.6 (2C), 121.0 (2C), 70.8, 57.5. LC-MS (APCI$^+$/ESI): *m/z* = 312.1 $[M+H]^+$, calculated exact mass for $C_{17}H_{16}BNO_5$: 311.10. Purity >99%, t_R = 1.021 min.

Methyl(R)-2-(1-hydroxy-1,3-dihydrobenzo[c][1,2]oxaborole-6-carboxamido)-2-phenylacetate, **30**

Obtained as a white solid (32 mg, 29%). R*f* (DCM:MeOD, 8:2) 0.35; m.p: 210-212 °C; ^{1}H NMR (400 MHz, MeOD) δ 7.93 (s, 1H), 7.65 (d, *J* = 7.7 Hz, 1H), 7.40 (d, *J* = 7.8 Hz, 2H), 7.17 – 7.08 (m, 3H), 5.40 (s, 1H), 2.30 (s, 3H). ^{13}C NMR (101 MHz, MeOD) δ 177.0, 170.3, 154.7, 139.1, 137.7, 132.7, 130.4, 129.8, 128.8, 128.2, 126.5 (2C), 121.3 (2C), 70.2, 60.6, 21.2. LC-MS (APCI$^+$/ESI): *m/z* = 326.1 $[M+H]^+$, calculated exact mass for $C_{17}H_{16}BNO_5$: 325.11. Purity >99%, tR = 2.206 min.

2-(3-chlorophenyl)-2-(1-hydroxy-1,3-dihydrobenzo[c][1,2]oxaborole-6-carboxamido)acetic acid, **32**

Obtained as a white solid (70 mg, 76%). m.p: 143-145 °C; R_f (DCM:MeOH, 9:1) 0.4; ^{1}H NMR (400 MHz, DMSO-d_6) δ 9.35 (s, 1H), 9.14 (d, *J* = 7.5 Hz, 1H, -NH), 8.27 (s, 1H), 7.99 (d, *J* = 7.9 Hz, 1H), 7.59 (s, 1H), 7.53 – 7.36 (m, 4H), 5.62 (d, *J* = 7.5 Hz, 1H), 5.05 (s, 2H). ^{13}C NMR (101 MHz, DMSO-d_6) δ 171.4, 166.6, 157.2, 139.9, 132.9, 132.7, 130.3, 130.1, 130.1, 127.9, 127.8 127.03, 121.3 (2C), 69.9, 56.4. LC-MS (APCI$^+$/ESI): *m/z* = 346.1 $[M+H]^+$, calculated exact mass for $C_{16}H_{13}BClNO_5$: 345.06. Purity = 97%, t_R = 2.227 min.

2-(3-fluorophenyl)-2-(1-hydroxy-1,3-dihydrobenzo[c][1,2]oxaborole-6-carboxamido)acetic acid, **33**

Obtained as a light yellow solid (39 mg, 47%). m.p: 132-135 °C; R_f (DCM:MeOH, 9:1) 0.4; ^{1}H NMR (400 MHz, DMSO-d_6) δ 9.34 (s, 1H), 9.17 (d, J = 7.6 Hz, 1H, -NH), 8.27 (s, 1H), 7.99 (d, J = 8.0 Hz, 1H), 7.60 – 7.46 (m, 2H, H4), 7.42 – 7.36 (m, 1H), 7.26 – 7.21 (m, 2H), 5.91 (d, J = 7.6 Hz, 1H), 5.04 (s, 2H). ^{13}C NMR (101 MHz, DMSO-d_6) δ 171.2, 166.6, 161.3, 158.9, 157.2, 132.6, 130.1, 130.1, 129.6, 124.5, 121.3 (2C), 115.5, 115.3, 69.9, 50.1. LC-MS (APCI$^+$/ESI): m/z = 330.1 [M+H]$^+$, calculated exact mass for $C_{16}H_{13}BFNO_5$: 329.09. Purity > 99%, t_R = 1.950 min.

Aqueous solubility

Solubility was performed using an adaptation of the miniaturised shake flask equilibrium solubility method.[1,2] 10mM stock solutions of the compounds in DMSO were transferred to 96-well plates using a Hamilton Microlab Starlet automated liquid handler. The DMSO was then dried down under vacuum in a Genevac Mivac Quattro centrifugal concentrator (90min, 37ºC). Thereafter, the samples were reconstituted (200 µM) in aqueous solution and shaken (200rpm, 20 hours, 25 °C). The same 10mM stocks were then used to prepare calibration standards (10-220 µM) in DMSO. The solutions were analysed by means of HPLC-DAD (Agilent 1200 Rapid Resolution HPLC with a diode array detector). Solubility was then determined using the peak areas of the aqueous samples and the best fit linear regression curves constructed using the calibration standards.

In vitro antiplasmodium Assay

The endpoint parasite lactate dehydrogenase assay

Compounds were tested using parasite lactate dehydrogenase as a marker for parasite survival as described.[3] Briefly, the respective stock solutions of CQ diphosphate and test compounds were prepared to 20 mg/mL in distilled water (for CQ) and 100% DMSO for test compounds and then stored at –20 °C, and further dilutions were prepared on the day of the experiment. The cultures were synchronized in the ring stage using 15 mL of 5% (w/v) D-sorbitol in water. Synchronous cultures of *Pf*NF54 (CQ-S) and *Pf*K1 (MDR) in the ring stage were prepared to 2% parasitemia & 2% hematocrit. Compounds were tested at starting concentrations of 10,000 ng/mL (1000 ng/mL for CQ), which were then serially diluted 2-fold in complete medium. An equivalent volume of parasite stock was added to each well. Parasites were incubated in the presence of the compounds at 37 °C under hypoxic conditions (4% CO_2 and 3% O_2 in N_2) for 72 h. After incubation, 100 µL of MalStat reagent and 15 µL of resus-

pended culture were combined in a duplicate plate, followed by addition of 25 µL of nitro blue tetrazolium chloride solution. The plates were kept in the dark for 10 min in order to fully develop, after which absorbance was measured at 620 nm on a microplate reader. Non-linear regression analysis was carried out using GraphPad Prism 4.0 (La Jolla, California, USA) to determine IC_{50} values.

The SYBR Green I assay

Asexual *Pf*NF54 (drug susceptible) and *Pf*K1 (drug resistant) parasite strains were cultivated under previously described conditions.[4] Parasites were maintained at 5% hematocrit (A^+/O^+ human erythrocytes) in complete culture media [RPMI 1640 (Sigma-Aldrich) supplemented with 25 mM HEPES, 20 mM D-glucose, 200 µM hypoxanthine, 0.2% (w/v) sodium bicarbonate, 24 µg/mL gentamicin, and 0.5% (w/v) AlbuMAX II)] and kept under hypoxic conditions (90% N_2, 5% O_2, and 5% CO_2) at 37 °C with agitation. D-sorbitol [5% (w/v)] was used to synchronize parasite cultures, resulting in predominantly (>95%) ring stages, when required. Parasite progression and morphology of asexual blood stages were monitored microscopically using Giemsa-stained thin smears.

The SYBR Green I assay enabled analysis of synchronized *in vitro Pf*NF54 and *Pf*K1 parasites (1% parasitemia, 1% hematocrit) exposed to drug pressure for 72 h at 90% N_2, 5% O_2, and 5% CO_2 with chloroquine as the positive control for inhibition of parasite proliferation. Parasite proliferation was determined once the 72 h incubation had elapsed by adding equal volumes of parasite suspension and SYBR Green I lysis buffer (0.2% µL/ml of 10 000x SYBR Green I (Invitrogen), 20 mM Tris-HCl, pH 7.5, 5 mM EDTA, 0.008% saponin (w/v) and 0.08% Triton x-100) and incubated for 1 h at room temperature in the dark. Fluorescence was quantified with the GloMaxR- Multi+Detection System at 485/538 nm and used as a direct measure of proliferation. Data was performed for three independent biological replicates of technical triplicates were performed for dose−response evaluation.

In vitro activity against gametocyte stage

The *in vitro* gametocyte activity was investigated in luciferase reporter line-based assay of the transgenic *Pf*NF54. In this luciferase assay a transgenic parasite line NF54-*Pf*S16-GFP-Luc was used to enable stage-specific assessment of gametocytocidal activity. Gametocytes were prepared according to the procedure reported by Reader *et al.*[4] Experiments were performed on days 5 (representing >90% of immature stage I – III gametocytes) and 10 (representing >90% late stage IV/V gametocytes). In each experiment, assays were set up with the use of 2 to 3% gametocytaemia, 1.5% haematocrit culture and 48 h drug pressure in a gas chamber (90% N_2, 5% O_2, and 5% CO_2) and temperature maintained at 37 °C. Luciferase activity was determined in 30 µL parasite lysates by adding 30 µL

luciferin substrate (Promega Luciferase Assay System) at room temperature and detection of resultant bioluminescence at an integration constant of 10s with the GloMax®-Multi+ Detection System with Instinct® Software. Methylene blue (5 µM) and MMV390048 (5 µM) were included as controls. The dual point screens were performed as technical triplicates for a single biological assay.

Table S1: *In vitro* gametocytocidal activity of selected analogues.

Compound	[a]*Pf*iGc IC_{50} (µM)	[b]*Pf*lGc IC_{50} (µM)
**10**	>20	>20
**31**	>20	15.9
**32**	>20	15.3
**33**	3.95	4.46

[a]IC_{50} determination of immature-stage (iGc, >90% I – III) and [b]late-stage (lGc, > 90% IV – V) gametocytes (n = 3, technical triplicates). Methylene blue (iGc luc at 1.0 µM = 95% inhibition, iGc IC_{50} = 0.2 µM; lGc luc at 1.0 µM = 92% inhibition, LGc IC_{50} = 0.14 µM). [c]Data generated using the Luciferase reporter assay. [d]Not determined. [e]n = 3, technical triplicates ± SEM.

In vitro cytotoxicity Assays

In vitro cytotoxicity evaluation against Chinese Hamster Ovarian (CHO) cell line

The *in vitro* cytotoxicity of selected synthesized compounds was evaluated against the CHO cell line using the MTT [3-(4,5-dimethylthiazolyl-2)-2,5- diphenyltetrazolium bromide] assay.[5] The compounds were assayed in three independent biological repeats in technical triplicates. Emetine was used as the reference standard. Stock solutions of 20 mg/mL of test compounds were prepared in DMSO and emetine was prepared to 2 mg/ml in distilled water. Compounds were tested in a dose-response evaluation from an initial concentration of 100 µg/mL. The CHO cells were incubated for 48 h with test compounds and developed afterward by adding 25 µl of sterile MTT solution to each well, followed by 4 h of incubation in the dark. The plates were then centrifuged, the medium aspirated, and 100 µL of DMSO was added to dissolve crystals before reading the absorbance at 540 nm. The cell viability was not affected by the highest concentration of the solvent to which the cells were exposed. Non-linear regression analysis was carried out using GraphPad Prism 4.0 (La Jolla, California, USA) to determine IC_{50} values.

In vitro hemolytic evaluation in red blood cells

Compounds were tested for hemolysis over 72h under the same conditions as the antiplasmodium assay, starting from 20 μM. Assay stocks were freshly prepared in complete medium from 10 mM compound stocks in DMSO. All wells in the plate were resuspended daily to ensure even mixing. After 72 h, wells were resuspended again and the plate centrifuged at 400g to 5 min. An aliquot the supernatant was transferred to a duplicate plate and read on a spectrophotometer at 540 nm. Readings were normalised against the supernatant from a freshly-prepared stock of human blood at 1% hematocrit in complete medium, representing 0% hemolysis, and the supernatant of a 1% stock of blood in complete medium which had undergone three rapid freeze-thaw cycles at -80/37 degrees, representing 100% hemolysis.

In vitro Metabolic Stability Assay

The metabolic stability assay was performed in duplicate in a 96-well micro titre plate with the test compounds (0.1 μM) were incubated (37 °C) in mouse, rat and pooled human liver microsomes (final protein concentration of 0.4 mg/mL; XenoTech, Lenexa, KS) suspended in 0.1 M phosphate buffer (pH 7.4) for 30 minutes, in the presence and absence of the cofactor NADPH (1 mM). The reactions were quenched by the addition of ice-cold acetonitrile containing an internal standard (carbamazepine 0.0236 μg/mL). The samples were centrifuged, and the supernatant was filtered and analysed by means of HPLC-MS/MS (Agilent Rapid Resolution HPLC, AB SCIEX 4500 MS). The relative loss of parent compound over time was monitored and plots were prepared for each compound of concentration versus time to determine the first order rate constant for compound depletion. This was used to calculate the degradation half–life and *in vitro* intrinsic clearance value.[6]

In vivo Antiplasmodium Assay (Swiss TPH)

In vivo efficacy was assessed as previously described,[7] with the modification that mice (n = 3) were infected with a GFP-transfected *P. berghei* ANKA strain (donated by A. P. Waters and C. J. Janse, Leiden University, The Netherlands), and parasitemia was determined using standard flow cytometry techniques. The detection limit was 1 parasite in 1000 erythrocytes (that is, 0.1%). The activity was calculated as the difference between the mean percent parasitemia for the control and treated groups expressed as a percent relative to the control group. Compounds were dissolved or suspended in a vehicle consisting of 70% Tween-80 and 30% ethanol, followed by a 10-fold dilution in H_2O and oral administration as four consecutive daily doses (4, 24, 48, and 72 h after infection). Blood samples for the quadruple-dose regimens were collected on day 4 (96 h after infection). The survival time in days was also recorded up to 30 days after infection. A compound was considered curative if the animal survived to day 30 after infection with no detectable parasites by slide reading.

In vivo studies conducted at the Swiss TPH, Basel were approved by the veterinary authorities of the Canton Basel-Stadt (Permit No. 1731) based on Swiss Cantonal (Verordnung Veterinäramt Basel-Stadt) and National Regulations (The Swiss Animal Protection Law, Tierschutzgesetz).

In vivo Pharmacokinetic Studies in Healthy Mice (H3D, DMPK)

Male Balb/c mice were used for animal pharmacokinetics evaluations. All animal work was carried out in accordance with the University of Cape Town's ethics policies using protocols approved by the research ethics committee (AEC022_00). The compounds were administered intravenously as a bolus of DMA/PEG/PPG:10/30/60) and orally in 0.5% (w/v) hydroxypropylmethylcellulose (HPMC) with 0.2% Tween-80 aqueous solution to male Balb/c mice (n = 3 for the oral group, and n = 3 for the intravenous group). Mice were not fasted overnight and were allowed to eat *ad libitum*. Animals were permitted access *ad libitum* to water.

PK Sampling: Blood samples were collected from the tail vein of the mice into heparinized micro-centrifugation tubes at predetermined sampling times (0.17, 0.5, 1, 3, 5, 7, 9, and 24 h for intravenous dosing; 0.5, 1, 3, 5, 7, 9, and 24 h for oral dosing). All samples were stored at −80 °C until extraction.

Bioanalytical Method:

Frozen whole blood was thawed at room temperature. Ten microliters of samples were extracted by protein precipitation using 100 µL of acetonitrile containing a 10 ng/mL internal standard (MMV394902) and centrifuged. Calibration standards and quality controls were extracted through the same procedure. Supernatants were injected onto the column for LC–MS/MS analysis. The analytical limit of quantitation (LOQ) was 2 ng/mL. The accuracy, precision, and recovery for each study were within acceptable limits.

Instrument	AB SCIEX 5500 QTRAP equipped with a Turbo V™ ion sourcecoupled to an Agilent 1260 HPLC.
Detection	Positive electrospray ionization under MRM scans
Column	Kinetex C18, 50 × 2.1 mm, 2.6 µM
LC Conditions	Gradient 0.4 ml/min, 7 min run, injection volume 5 µl, column temperature 40°C, sample tray temperature 8°C

Mobile Phase	A: 0.1% formic acid; B 0.1% formic acid in acetonitrile
Analysis Software	Analyst® 1.6.3 software for instrument control, data acquisition and analysis.

Calculation of Pharmacokinetics Parameters: PK parameters were calculated by noncompartmental analysis using PK Solutions 2.0 (Summit Research Services, Montrose, CO, USA) with a method based on curve stripping.

Barcoded Cross-resistance Studies

Cross-resistance using a pool of barcoded lines was assessed essentially as previously described.[8] The parasite pool of resistant mutants consisted of 45 barcoded lines (Table S2), generated via CRISPR/Cas9 genome-editing to integrate unique barcodes into a nonessential gene (*pfpare*/PF3D7_0709700), covering both the chloroquine-resistant Dd2 and chloroquine-sensitive 3D7 genetic backgrounds, including wild-type (WT) lines for both strains. The pool of freshly thawed mutant lines was exposed to compound **7** at 3 × IC_{50} for 14 days alongside an untreated control pool of the same lines grown without drug pressure. The proportions of mutant lines remaining in the treated vs untreated pools were measured via next generation sequencing to track the unique barcodes and quantified as the log2 of the fold change in proportions. The competitive outgrowth of any line with log2 fold change > 2.5 signified cross resistance, while those measuring < -2.5 showed a significant sensitivity to the compound.

Table S2: The list of lines/mutations, gene names and gene IDs

Line name	Gene Description	Gene ID	Input (%) Day 0	No drug (%) Day 14	7 (%) Day 14
3D7	Wild type		4.392	1.934	2.262
3D7 ABCI3 R2180P	ABC transporter family member 1	PF3D7_0319700	0.004	0.000	0.001
3D7 ACS10 M300I	Acyl CoA synthase	PF3D7_0525100	0.364	0.030	0.042
3D7 ACS11 D648Y	Acyl CoA synthase	PF3D7_1238800	0.002	0.000	0.000
3D7 ACS11 E668K	Acyl CoA synthase	PF3D7_1238800	0.005	0.000	0.000
3D7 ATP2 CNV2	Phospholipid-transporting ATP2	PF3D7_1219600	0.011	0.001	0.000
3D7 DHFR-TS G378E	Dihydrofolate reductase-	PF3D7_0417200	0.148	0.006	0.015

	thymidylate synthase				
3D7 DHFR-TS I403L	Dihydrofolate reductase-thymidylate synthase	PF3D7_0417200	0.172	0.010	0.016
3D7 FTb A515T	Farnesyltransferase subunit beta	PF3D7_1147500	0.473	0.027	0.053
3D7 MDR2 K840N	Multidrug resistance protein 2	PF3D7_1447900	0.025	0.000	0.002
3D7 NCR1 A1108T	Niemann-Pick type C1-related protein	PF3D7_0107500	0.091	0.009	0.014
Dd2	Wild type		17.973	46.447	46.526
Dd2 AcAS A597V	Acetyl-CoA synthetase	PF3D7_0627800	0.116	0.010	0.010
Dd2 ATP4 A353E CARL I1139K	ATP4+CARL	PF3D7_1211900	0.096	0.004	0.001
Dd2 ATP4 G358S	Non-SERCA-type Ca2+ -transporting P-ATPase (ATP4)	PF3D7_1211900	1.632	0.375	0.304
Dd2 CARL I1139K	Cyclic amine resistance locus (CARL)	PF3D7_0321900	0.166	0.031	0.024
Dd2 CARL L1073Q	Cyclic amine resistance locus (CARL)	PF3D7_0321900	0.072	0.004	0.010
Dd2 CARL V1103L	Cyclic amine resistance locus (CARL)	PF3D7_0321900	0.123	0.023	0.013
Dd2 CPSF Y408S (edit)	Cleavage and polyadenylation specific factor	PF3D7_1438500	0.013	0.001	0.001
Dd2 CPSF Y408S (sel)	Cleavage and polyadenylation specific factor	PF3D7_0709000	0.005	0.000	0.000
Dd2 CRT M343L	Chloroquine resistance transporter	PF3D7_1250200	0.585	0.164	0.165
Dd2 CSC1 L800P	CSC1-like protein putative	PF3D7_MIT02300	0.241	0.103	0.115
Dd2 cytBC1 G33V	Cytochrome b	PF3D7_MIT02300	0.202	0.022	0.029
Dd2 cytBC1 V284L	Cytochrome b	PF3D7_0417200	0.363	0.164	0.204
Dd2 DHFR-TS S216R	Dihydrofolate reductase-thymidylate synthase	PF3D7_0603300	1.430	0.666	0.831
Dd2 DHODH C276Y	Dihydroorotate dehydrogenase	PF3D7_0603300	4.095	5.987	6.026
Dd2 DHODH F227I	Dihydroorotate dehydrogenase	PF3D7_0603300	0.018	0.002	0.000
Dd2 DHODH I263F	Dihydroorotate dehydrogenase	PF3D7_1451100	0.016	0.003	0.002
Dd2 DHODH L531F	Dihydroorotate dehydrogenase	PF3D7_1451100	0.045	0.011	0.004
Dd2 eEF2 L755F	Elongation factor 2	PF3D7_1128400	1.192	1.265	1.454
Dd2 eEF2 Y186N	Elongation factor 2	PF3D7_1332900	0.034	0.000	0.001
Dd2 GGPPS S228T	Geranylgeranyl diphosphate synthase	PF3D7_1332900	0.304	0.051	0.047
Dd2 IleRS E180D	Ile-tRNA synthetase	PF3D7_1332900	7.306	4.420	4.151
Dd2 IleRS L810F	Ile-tRNA synthetase	PF3D7_1343700	5.528	4.728	4.862
Dd2 IleRS V500A	Ile-tRNA synthetase	PF3D7_1343700	18.130	12.422	11.289

Dd2 kelch13 C580C	Kelch protein K13	PF3D7_1343700	15.354	13.077	12.220
Dd2 kelch13 C580Y	Kelch protein K13	PF3D7_0908800	5.610	2.494	2.547
Dd2 kelch13 R539T	Kelch protein K13	PF3D7_0523000	0.820	0.317	0.347
Dd2 MDR1 F1072L	Multidrug resistance protein 1	PF3D7_0509800	0.066	0.012	0.013
Dd2 PI4K CNV	Phosphatidylinositol 4-kinase	PF3D7_0509800	2.490	1.612	1.865
Dd2 PI4K S1320L+L1418F	Phosphatidylinositol 4-kinase	PF3D7_0509800	7.300	1.930	2.604
Dd2 PI4K S743F+H1484Y	Phosphatidylinositol 4-kinase	PF3D7_1213800	1.985	1.339	1.577
Dd2 ProRS L482H	Pro-tRNA synthetase	PF3D7_1011400	0.398	0.221	0.317
Dd2 UDP-GT F37V	UDP-galactose transporter	PF3D7_1017000	0.044	0.004	0.001
Dd2-pol-delta D308A-E310A	DNA polymerase delta	PF3D7_1017000	0.559	0.073	0.034

Metabolomics

Parasite culture maintenance

Pf3D7 strain was maintained under standard culture conditions at 2% hematocrit with O-positive human erythrocytes according to the conditions described by Allman *et al.*[9] Ring-stage developing parasites were suspended in 5% sorbitol (Sigma-Aldrich) over three subsequent developmental cycles to synchronize parasites within 4 to 6 h, as previously described. *P. falciparum* cultures were collected by centrifugation at 1,500 × g for 5 min at 25 °C, and the parasitized RBC pellet was resuspended in a 10 × volume of 5% sorbitol, followed by incubation at 37 °C for 10 min. Following incubation, the cells were pelleted and washed in 50 ml of complete medium, prior to placement of the parasites back into culture flasks at 2% hematocrit.

Sample preparation for metabolomics

Hydrophilic metabolite changes following compound intervention were profiled according to the procedure by Allman *et al.*[9] Treatments were performed on 1 × 10^8 MACS-purified, synchronized trophozoite parasite-infected RBCs (24-36 h post invasion) in 5 mL RPMI. Compounds were added at a concentration of 10 × IC_{50} and incubated for 2.5 h. All treatment conditions were performed as technical triplicates and included an untreated control with an atovaquone control in every set-up. Subsequently, PBS washes were performed, and infected RBCs were extracted with 90% methanol containing 0.5 µM $^{13}C^{15}N$-labelled aspartate as an internal standard, then dried under nitrogen and stored at -80 °C. Process blanks were generated at the time of extraction in technical triplicates. Sam-

ples were then resuspended in high-performance liquid chromatography (HPLC) grade water containing 1 µM chlorpropamide as an additional internal standard and analyzed by ultra-high-performance liquid chromatography mass spectrometry UHPLC-MS as described.[9,10]

Following negative ionization analysis of hydrophilic extracts on a Thermo Exactive Plus Orbitrap, sample data were converted and transferred for analysis. Targeted peak picking from a curated list of 298 metabolites was achieved using el-MAVEN software[11], followed by normalization and analysis via RStudio and Metaboanalyst.[12] Data visualization was performed with the Hyperspec (http://hyperspec.r-forge.r-project.org) and Suprahex R[13] scripting packages in RStudio. Metabolomic profiling through hierarchical clustering for identification of related metabolic signatures was performed using the Ward method, based on the Pearson correlation coefficients, by the Hyperspec R integrated heatmap function.

Spectra data of key compounds

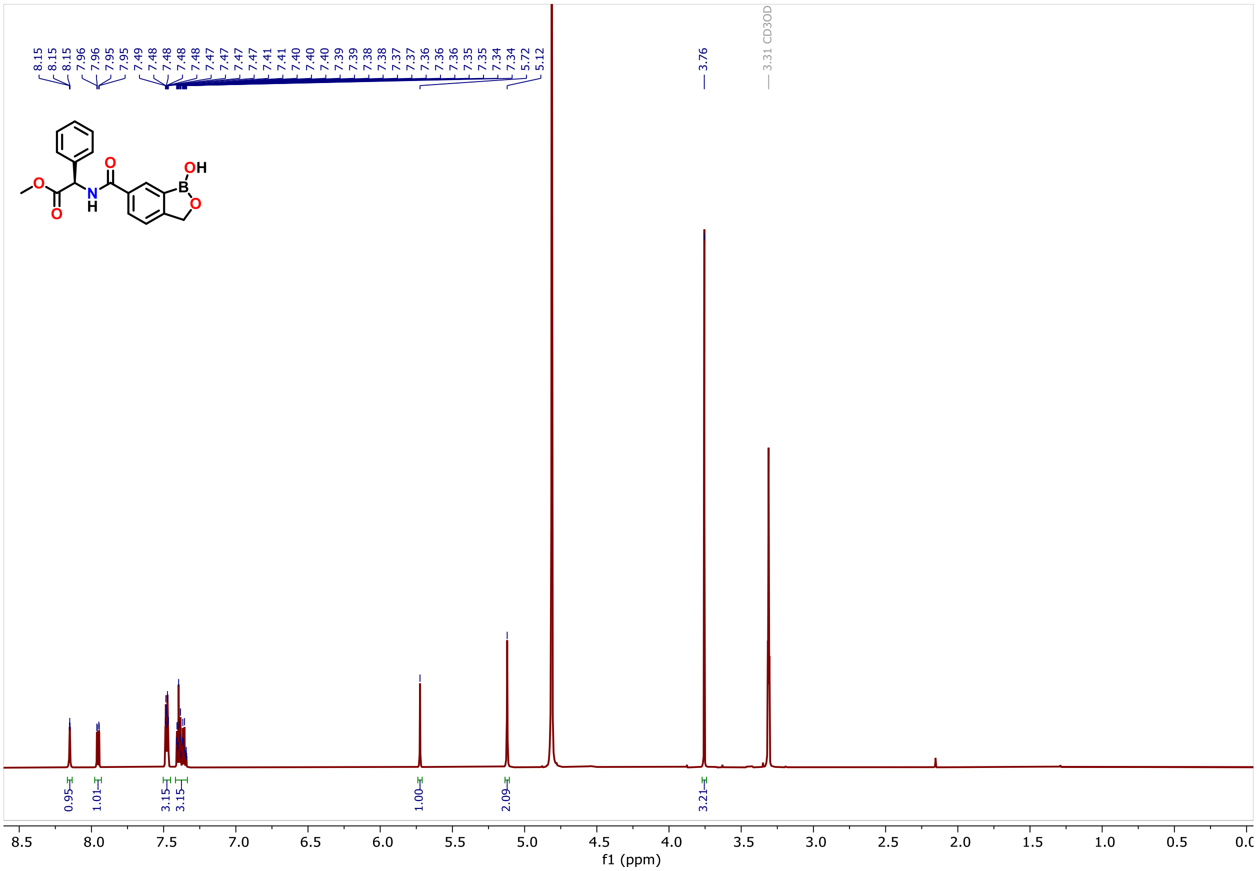

Figure S1: ^{1}H-NMR Spectrum of Compound **6** in Methanol-d_4 at 400 MHz

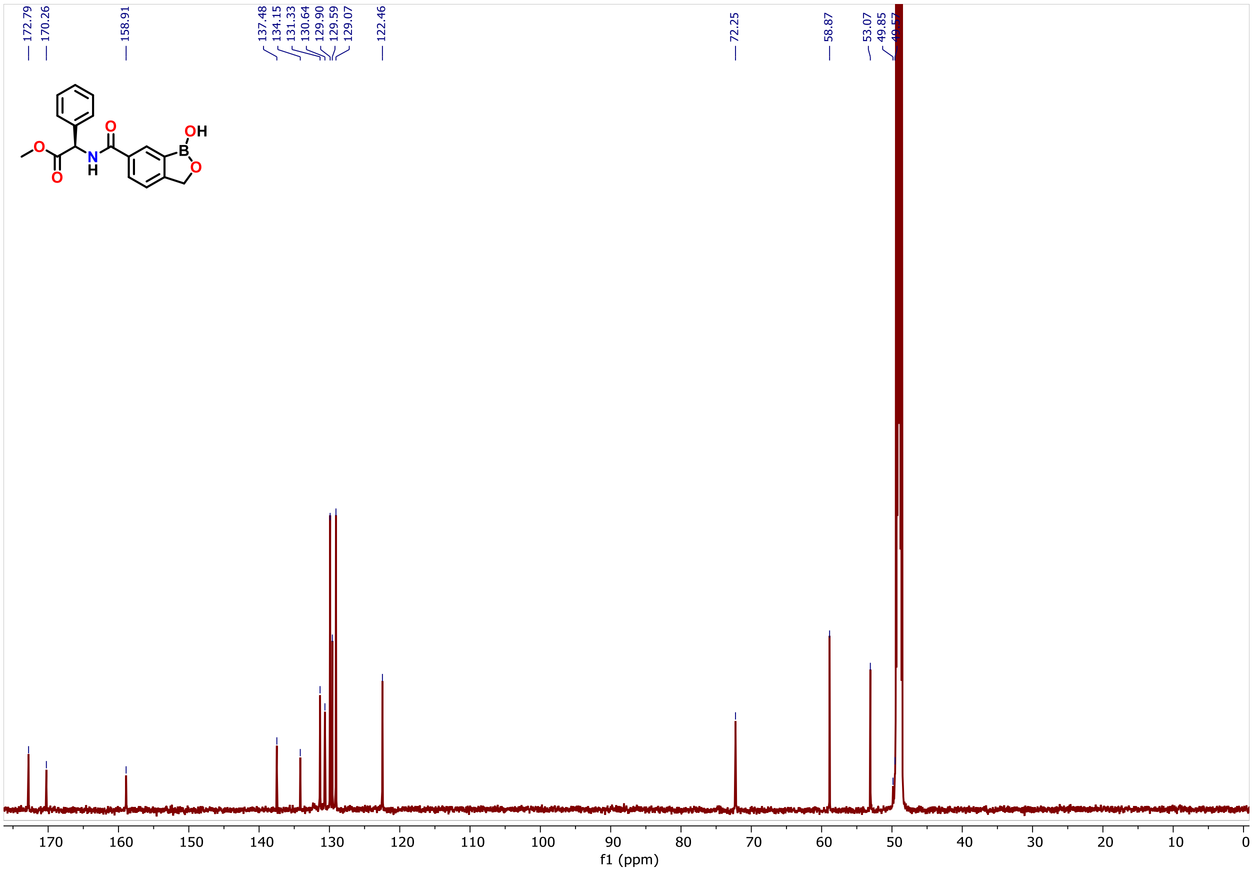

Figure S2: ^{1}H-NMR Spectrum of Compound **6** in Methanol-d_4 at 400 MHz

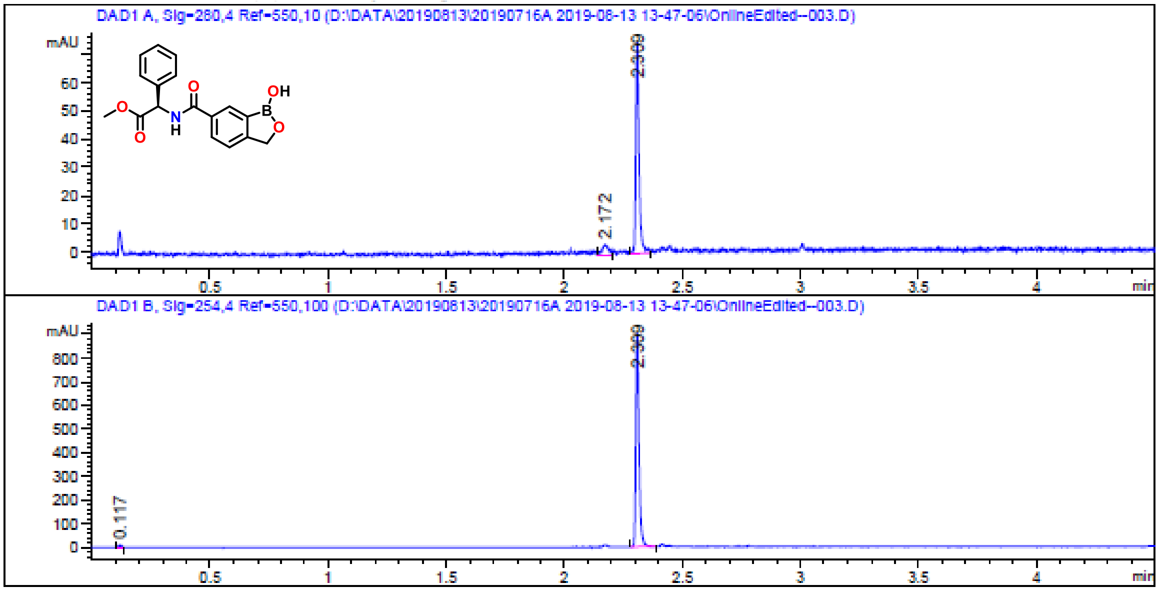


```
=====================================================================
                         Area Percent Report
=====================================================================

Sorted By             :      Signal
Multiplier            :      1.0000
Dilution              :      1.0000
Do not use Multiplier & Dilution Factor with ISTDs
```

Signal 1: DAD1 A, Sig=280,4 Ref=550,10

Peak #	RetTime [min]	Type	Width [min]	Area [mAU*s]	Height [mAU]	Area %
1	2.172	VV	0.0219	6.23227	3.56996	7.8687
2	2.309	BV	0.0150	72.97066	73.76673	92.1313

Totals : 79.20293 77.33669

Signal 2: DAD1 B, Sig=254,4 Ref=550,100

Peak #	RetTime [min]	Type	Width [min]	Area [mAU*s]	Height [mAU]	Area %
1	0.117	BV	0.0131	8.59908	10.44819	0.9769
2	2.309	BB	0.0149	871.61951	895.24634	99.0231

Totals : 880.21859 905.69453

Figure S3: LC Spectrum of Compound of **6**

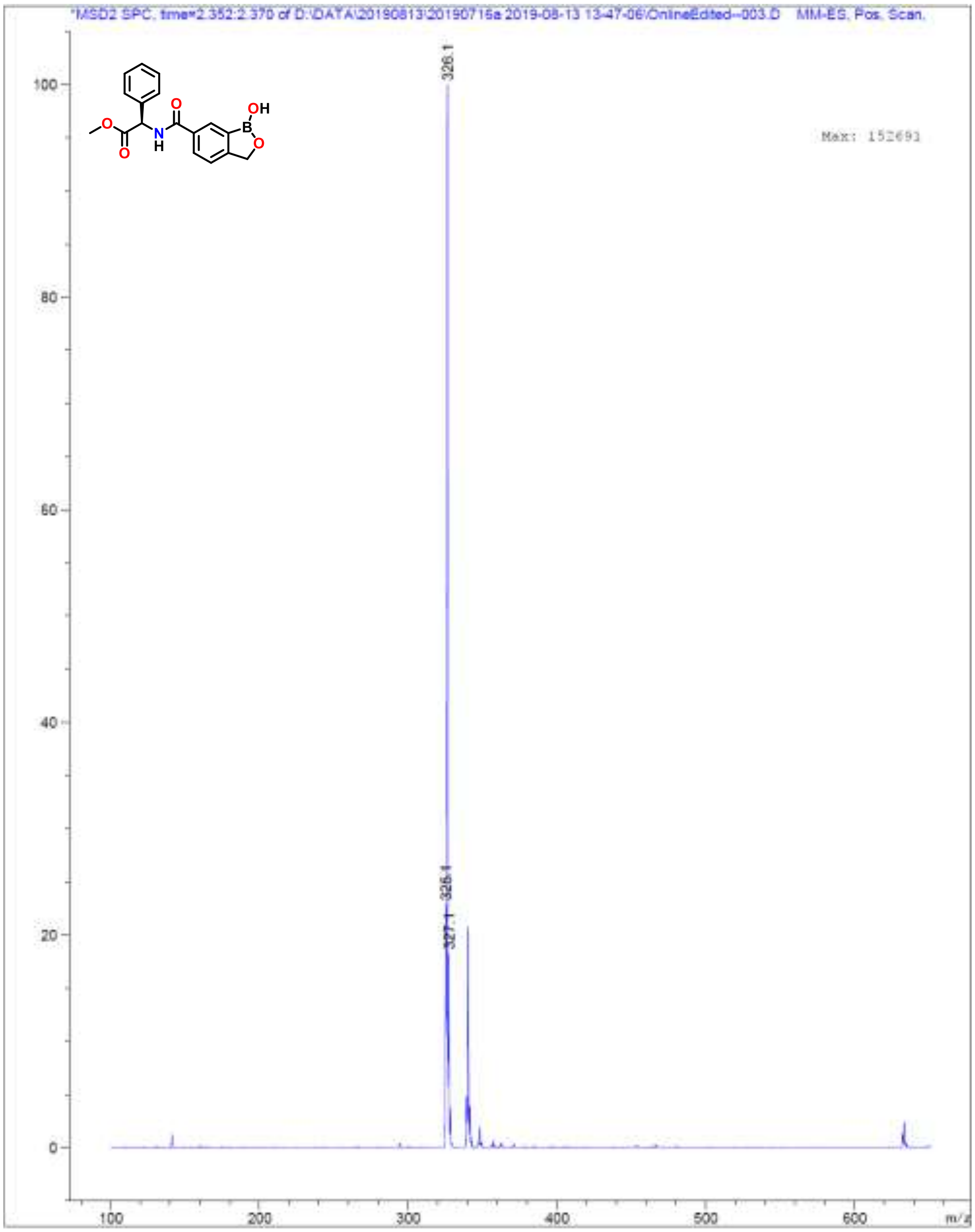

Figure S4: MS of Compound of **6**

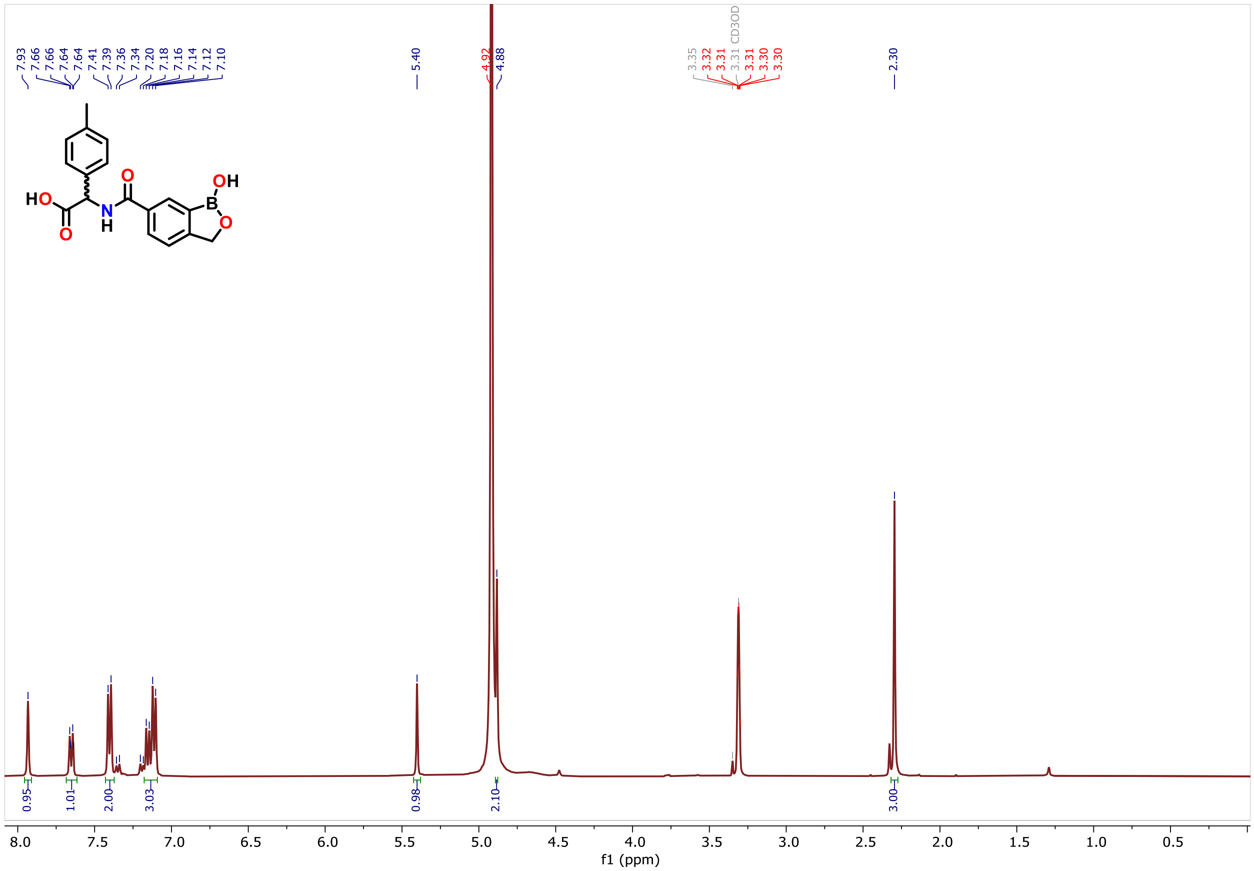

Figure S5: ^{1}H-NMR Spectrum of Compound **30** in Methanol-d_4 at 400 MHz

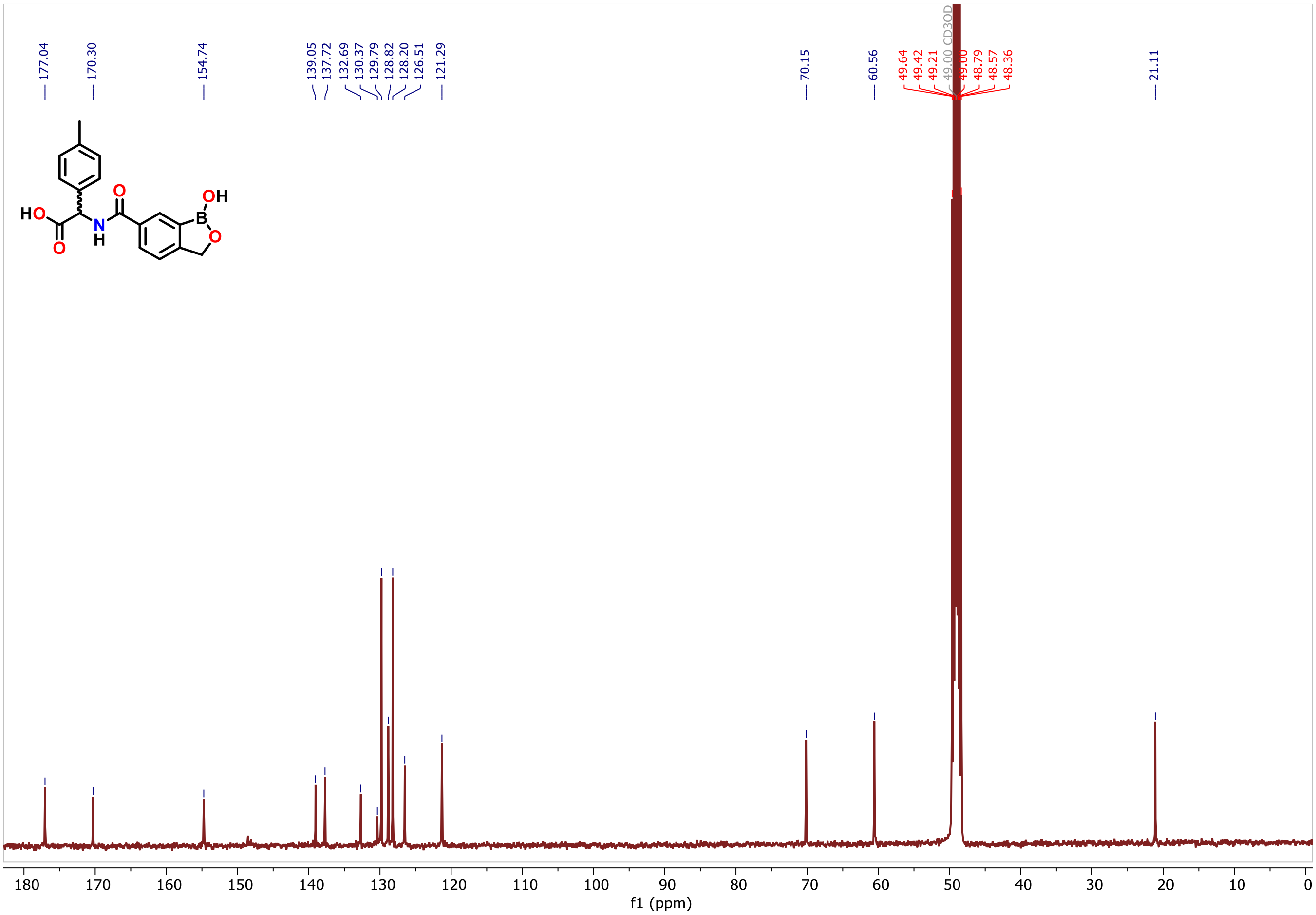

Figure S6: ^{13}C-NMR Spectrum of Compound **30** in Methanol-d_4 at 400 MHz

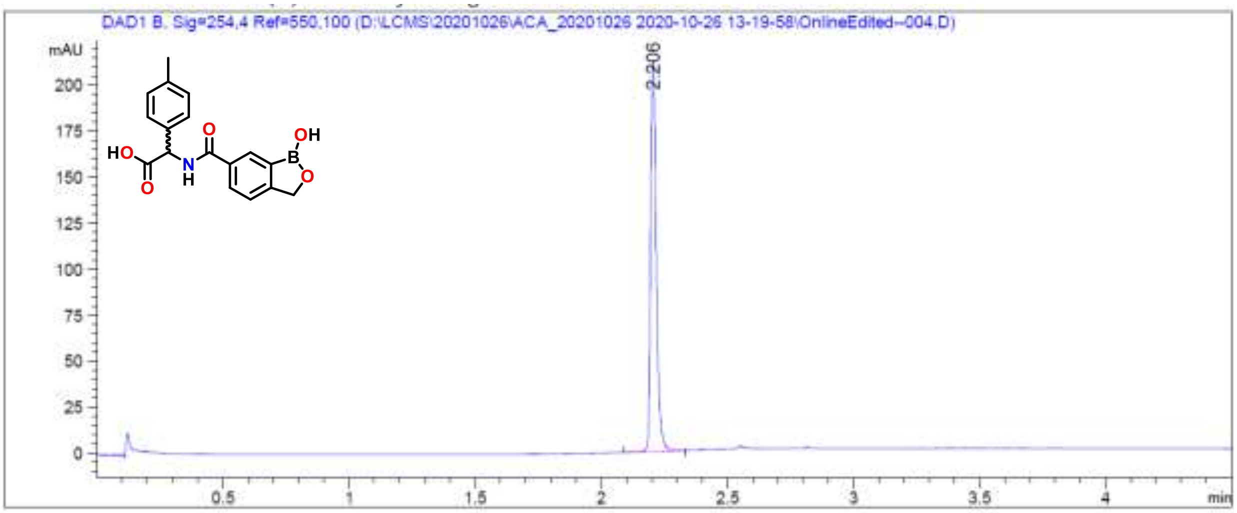


```
=====================================================================
                         Area Percent Report
=====================================================================

Sorted By             :      Signal
Multiplier            :      1.0000
Dilution              :      1.0000
Do not use Multiplier & Dilution Factor with ISTDs


Signal 1: DAD1 B, Sig=254,4 Ref=550,100

Peak RetTime Type  Width     Area      Height     Area
  #   [min]        [min]   [mAU*s]     [mAU]        %
----|-------|----|-------|----------|----------|--------|
   1   2.206 BV    0.0244  336.00613  212.58795 100.0000

Totals :                   336.00613  212.58795
```

Figure S7: LC Spectrum of Compound **30**

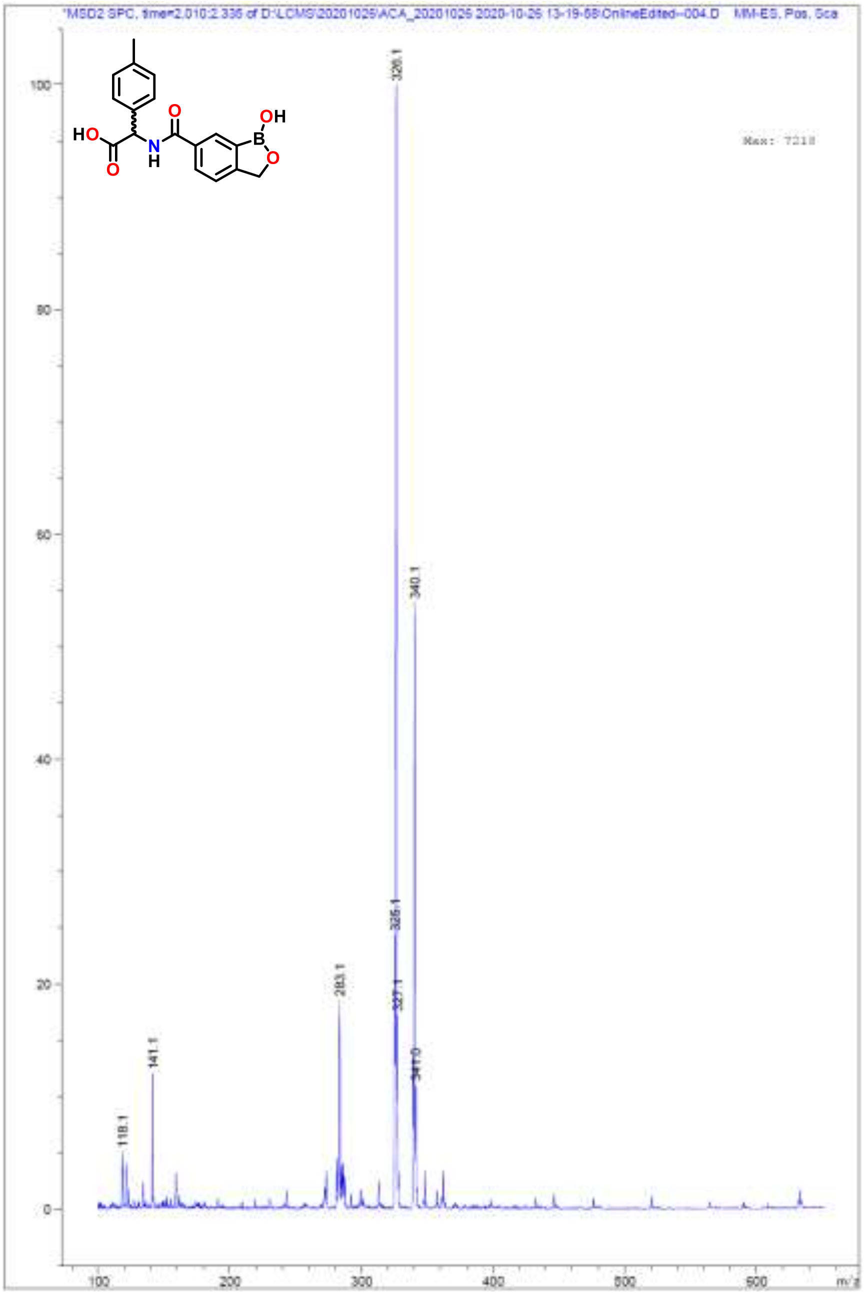

Figure S8: MS of Compound **30**

References

(1) Alelyunas, Y. W.; Liu, R.; Pelosi-Kilby, L.; Shen, C. Application of a Dried-DMSO rapid throughput 24-h equilibrium solubility in advancing discovery candidates. *Eur. J. Pharm. Sci.* **2009**, *37* (2), 172-182.

(2) Zhou, L.; Yang, L.; Tilton, S.; Wang, J. Development of a high throughput equilibrium solubility assay using miniaturized shake-flask method in early drug discovery. *J. Pharm. Sci.* **2007**, *96* (11), 3052-3071.

(3) Makler, M.; Ries, J.; Williams, J.; Bancroft, J.; Piper, R.; Gibbins, B.; Hinrichs, D. Parasite lactate dehydrogenase as an assay for *Plasmodium falciparum* drug sensitivity. *Am. J. Trop. Med. Hyg.* **1993**, *48* (6), 739-741.

(4) Reader, J.; Botha, M.; Theron, A.; Lauterbach, S. B.; Rossouw, C.; Engelbrecht, D.; Wepener, M.; Smit, A.; Leroy, D.; Mancama, D.; *et al.* Nowhere to hide: interrogating different metabolic parameters of *Plasmodium falciparum* gametocytes in a transmission blocking drug discovery pipeline towards malaria elimination. *Malar. J.* **2015**, *14*, 1-17.

(5) Liu, Y.; Peterson, D. A.; Kimura, H.; Schubert, D. Mechanism of cellular 3-(4, 5-dimethylthiazol-2-yl)-2, 5-diphenyltetrazolium bromide (MTT) reduction. *J. Neurochem.* **1997**, *69* (2), 581-593.

(6) Bertrand, M.; Jackson, P.; Walther, B. Rapid assessment of drug metabolism in the drug discovery process. *Eur. J. Pharm. Sci.* **2000**, *11*, S61-S72.

(7) González Cabrera, D.; Douelle, F.; Younis, Y.; Feng, T.-S.; Le Manach, C.; Nchinda, A. T.; Street, L. J.; Scheurer, C.; Kamber, J.; White, K. L.; *et al.* Structure–activity relationship studies of orally active antimalarial 3, 5-substituted 2-aminopyridines. *J. Med. Chem.* **2012**, *55* (24), 11022-11030.

(8) Carrasquilla, M.; Drammeh, N. F.; Rawat, M.; Sanderson, T.; Zenonos, Z.; Rayner, J. C.; Lee, M. C. Barcoding genetically distinct Plasmodium falciparum strains for comparative assessment of fitness and antimalarial drug resistance. *Mbio* **2022**, *13* (5), e00937-00922.

(9) Allman, E. L.; Painter, H. J.; Samra, J.; Carrasquilla, M.; Llinás, M. Metabolomic profiling of the malaria box reveals antimalarial target pathways. *Antimicrob. Agents Chemother.* **2016**, *60* (11), 6635-6649.

(10) Murithi, J. M.; Owen, E. S.; Istvan, E. S.; Lee, M. C.; Ottilie, S.; Chibale, K.; Goldberg, D. E.; Winzeler, E. A.; Llinás, M.; Fidock, D. A.; *et al.* Combining stage specificity and metabolomic profiling to advance antimalarial drug discovery. *Cell Chem. Biol.* **2020**, *27* (2), 158-171.

(11) Agrawal, S.; Kumar, S.; Sehgal, R.; George, S.; Gupta, R.; Poddar, S.; Jha, A.; Pathak, S. El-MAVEN: a fast, robust, and user-friendly mass spectrometry data processing engine for metabolomics. *Methods Mol. Biol.* **2019**, *1978*, 301-321.

(12) Chong, J.; Soufan, O.; Li, C.; Caraus, I.; Li, S.; Bourque, G.; Wishart, D. S.; Xia, J. MetaboAnalyst 4.0: towards more transparent and integrative metabolomics analysis. *Nucleic Acids Res.* **2018**, *46* (1), 486-494.

(13) Fang, H.; Gough, J. supraHex: an R/Bioconductor package for tabular omics data analysis using a supra-hexagonal map. *Biochem. Biophys. Res. Commun.* **2014**, *443* (1), 285-289.
